# IFIT3-DVL interaction promotes malignant progression of lung squamous cell carcinoma and large-cell lung carcinoma *via* canonical WNT signaling

**DOI:** 10.1016/j.jbc.2026.111368

**Published:** 2026-03-16

**Authors:** Yudie Lu, Mengdi Yang, Jingrong Zheng, Di Zhang, Qiang Han, Xinran Zhao, Minjia Li, Ruoqi Zhao, Guangping Wu, Enhua Wang, Huanyu Zhao

**Affiliations:** 1 Department of Pathology, The First Hospital and College of Basic Medical Sciences, China Medical University, Shenyang, Liaoning, P.R. China; 2 Basic Medicine, China Medical University, Shenyang, Liaoning, P.R. China; 3 8-Year Program, China Medical University, Shenyang, Liaoning, P.R. China

**Keywords:** canonical WNT signaling, dishevelled2, Interferon-induced protein with tetratricopeptide repeat 3, large-cell lung carcinoma, lung squamous cell carcinoma

## Abstract

Interferon-induced protein with tetratricopeptide repeat 3 (IFIT3) is involved in malignant progression. However, little information is available regarding its expression and detailed mechanisms in lung cancer. Herein, the clinicopathological significance of IFIT3 expression in lung squamous cell carcinoma (LUSC) and large-cell lung carcinoma (LCLC) specimens was assessed. IFIT3-overexpression and IFIT3-knockout LUSC/LCLC cells were generated both *in vitro* and *in vivo*. IFIT3 overexpression is correlated with advanced tumor-node-metastasis stage, lymph node metastasis, and poor prognosis in patients with LUSC and LCLC. IFIT3 promotes the malignant phenotypes of LUSC/LCLC cells *in vitro* and *in vivo*. The interaction between IFIT3 and dishevelled (DVL) in the cytoplasm of LUSC/LCLC cells was identified. Among DVL isoforms (DVL1, DVL2, DVL3), IFIT3-DVL2 interaction most prominently activates canonical wingless-type MMTV integration site family (WNT) signaling. This interaction promotes the phosphorylation of DVL2 at threonine 224 to increase the phosphorylation levels of glycogen synthase kinase-3β at serine nine and β-catenin at serine 675 and the expression of active β-catenin. Consequently, β-catenin nuclear translocation is elevated to activate β-catenin/TCF mediated transcription and upregulate the expressions of target genes of canonical WNT pathway (Cyclin D1, c-MYC, AXIN2) and the protein factors related to cell malignancy (CDK4/6, CDC42, MMP2/7/9). DVL2 knockdown or XAV-939 significantly abrogates above effects mediated by IFIT3 (*p* < 0.05). Overall, we demonstrated a novel signal transduction pathway where IFIT3 interacts with DVL2 to stabilize cytosolic β-catenin and promote β-catenin nuclear translocation *via* DVL2 phosphorylation, enhancing canonical WNT signaling activity and providing a potential target for clinical intervention in LUSC and LCLC.

Lung cancer is the leading cause of cancer-related deaths worldwide, with non-small cell lung cancer (NSCLC) accounting for approximately 85% of all malignant pulmonary tumors. NSCLC includes lung adenocarcinoma (LUAD), lung squamous cell carcinoma (LUSC), and large-cell lung carcinoma (LCLC) (1, 2). The late-stage identification of lung cancer often results in poor patient prognosis. Recent advancements in molecular biology research on pulmonary malignancies have opened new avenues for understanding the pathogenesis and identifying novel therapeutic targets.

Interferon-induced protein with tetratricopeptide repeat (IFIT) family of proteins have a tetratricopeptide repeat (TPR) motif. IFIT3, an important member of this family, locates in the cytoplasm and lacks known enzymatic function. It mediates multiple cellular functions by facilitating protein-protein interactions and forming multiprotein complexes with cellular factors *via* the TPR motif (3, 4). The TPR arranges in a helix-turn-helix conformation, with adjacent TPR motifs packed in a parallel fashion (5). This conformation is characterized by an uneven surface and facilitates the interaction with other protein factors (6). Protein factors containing TPR motifs can form various complexes with other protein factors in a series of signaling pathways (7). Although IFIT3 has a simple genetic and promoter structure, it performs a variety of complex functions, depending on the cell and tissue type. Chemokines derived from cancer-associated fibroblasts enhance the migration and metastasis mediated by IFIT3 in hepatocellular carcinoma cells (8). Additionally, patients with pancreatic ductal adenocarcinoma exhibiting upregulated IFIT3 expression show increased resistance to chemotherapy (9).

IFIT3, also known as retinoic acid-induced gene G (Rig-G) (10), acts as a cancer suppressor gene in the human LUAD cell line A549, by activating the p53 signaling pathway (11), whereas overexpression of collagen type VIII alpha one chain contributes to LUAD progression through EGFR activation induced by elevated IFIT3 expression (12). However, the expression patterns of IFIT3 in LUSC and LCLC specimens and the detailed mechanism of action of IFIT3 in the malignant progression of LUSC and LCLC remain underexplored. Therefore, we aim to explore this issue.

In this study, we detected the expression of IFIT3 in LUSC and LCLC specimens and analyzed its clinical significance. We also tested the hypothesis that IFIT3 affects the malignant progression of LUSC and LCLC cells through the WNT/β-catenin pathway.

## Results

### IFIT3 overexpression in LUSC and LCLC is associated with a poor prognosis

**Figure 1:**
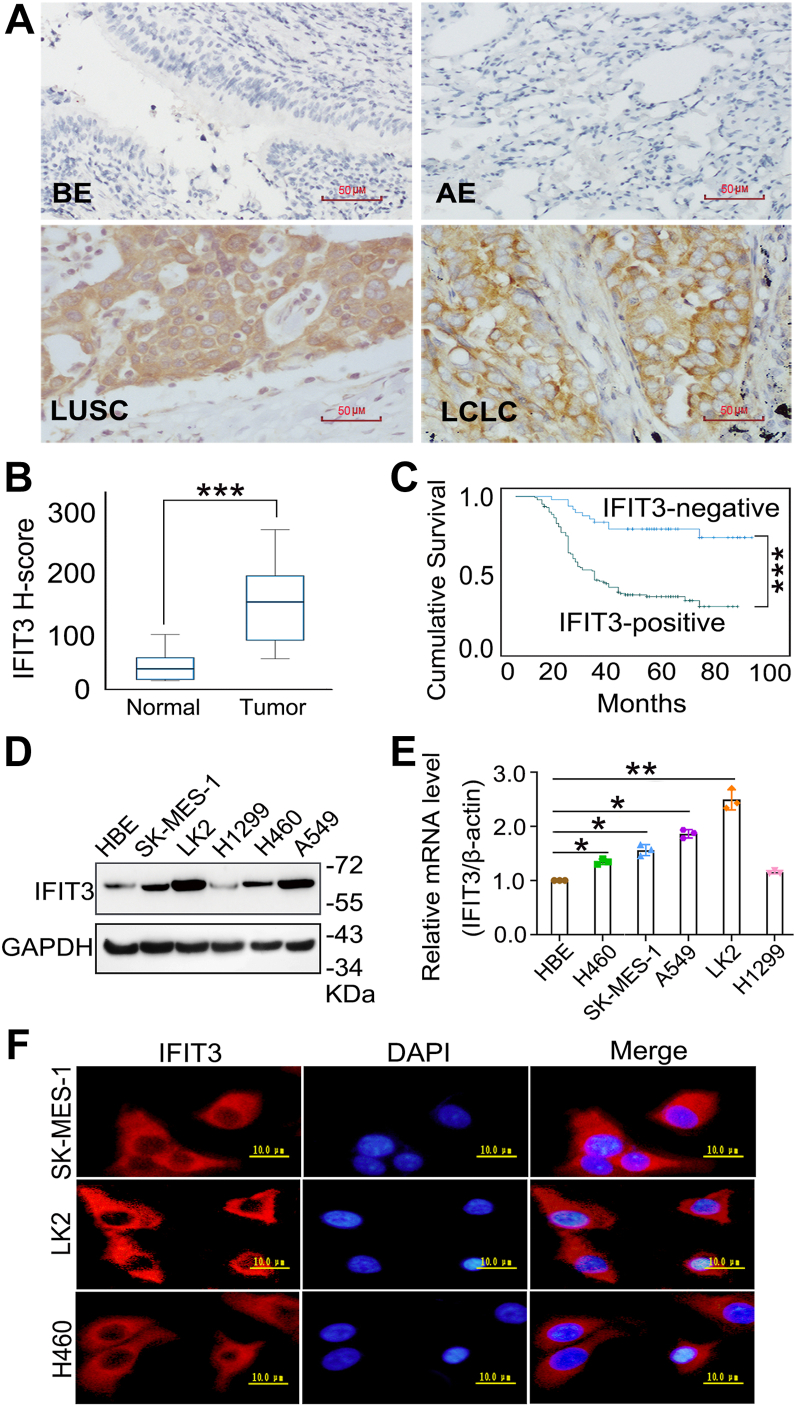
**IFIT3 expression in LUSC/LCLC tissues and cell lines.***A*, representative immunohistochemistry images of IFIT3 staining in normal bronchial epithelial, alveolar epithelial, LUSC, and LCLC (magnification: × 400; the scale bar represents, 50 μm), with an H-score (*B*) of NSCLC tissues vs. adjacent nontumorous tissues; NSCLC tissues (Tumor) include LUSC and LCLC, and adjacent nontumorous tissues (Normal) include bronchial epithelial and alveolar epithelial. *C*, survival duration of patients showing IFIT3-negative immunostaining and IFIT3-positive immunostaining. *D*, IFIT3 protein expression in five NSCLC cell lines and the normal bronchial cell line HBE. *E*, IFIT3 mRNA expression in five NSCLC cell lines and HBE cell line (comparing each NSCLC cell line with HBE cell line). *F*, representative immunofluorescence images of IFIT3 localization (the scale bar represents, 10 μm). Error bars indicated mean ± SEM (n = 3). Log-rank test was used for survival comparison. Data were analyzed by two-tailed unpaired Student’s *t* test for (*B*) and one-way ANOVA with Dunnett’s *post hoc* test for (*E*). ∗*p* < 0.05, ∗∗*p* < 0.01, ∗∗∗*p* < 0.001. HBE, human bronchial epithelial; LCLC, large-cell lung carcinoma; NSCLC, non-small cell lung cancer.

IFIT3 expression was assessed in 182 NSCLC samples (166 LUSC and 16 LCLC specimens) and 36 paired non-cancerous specimens were detected using immunohistochemical staining. Negative IFIT3 expression was observed in normal bronchial and alveolar epithelial cells, whereas 74.7% of NSCLC tissue samples (136/182) showed positive IFIT3 expression (Fig. 1*A*, Table 1). Expression was consistently higher in LUSC/LCLC cells than in normal bronchial and alveolar epithelial cells in each subgroup of IFIT3 status, with a mean H-score of 141.92 ± 4.746 vs. 27.28 ± 4.201 (Fig. 1*B*; *p* < 0.05). IFIT3 overexpression significantly correlated with lymph node metastasis and advanced tumor-node-metastasis (TNM) stage in patients with LUSC and LCLC (Table 1, *p* < 0.05). Kaplan-Meier estimation of survival curves demonstrated that patients with LUSC/LCLC exhibiting positive IFIT3 expression had significantly shorter overall 5-years survival rates than those with negative IFIT3 expression (Fig. 1*C*; *p* < 0.05).

**Table 1:** IFIT3 expression in relation to clinicopathological variables

Variables	Total	IFIT3 expression		*p*
		Negative	Positive	
	182	46	136	
Age (years)				
<60	70	21	49	0.246
≥60	112	25	87	
Gender				
Male	102	21	81	0.100
Female	80	25	55	
Histology				
LUSC	166	43	123	0.529
LULC	16	3	13	
Tumor-node-metastasis stage				
I	79	29	50	0.002
II + III	103	17	86	
Lymphatic metastasis				
No	71	26	45	0.005
Yes	111	20	91	
Differentiation				
Well	72	21	51	0.328
Moderate or poor	110	25	85	

IFIT3 expression was detected in various NSCLC cells using Western blot, quantitative real-time PCR, and immunofluorescence. IFIT3 showed higher expression in LUSC and LCLC cells than in the normal human bronchial epithelial cell line human bronchial epithelial (Fig. 1, *D* and *E*, *p* < 0.05). Most IFIT3 located in the cytoplasm of these cells (Fig. 1*F*). Collectively, IFIT3 overexpression is a clinical marker of poor prognosis in LUSC and LCLC.

### IFIT3 promotes the malignant phenotype of LUSC and LCLC cells

**Figure 2:**
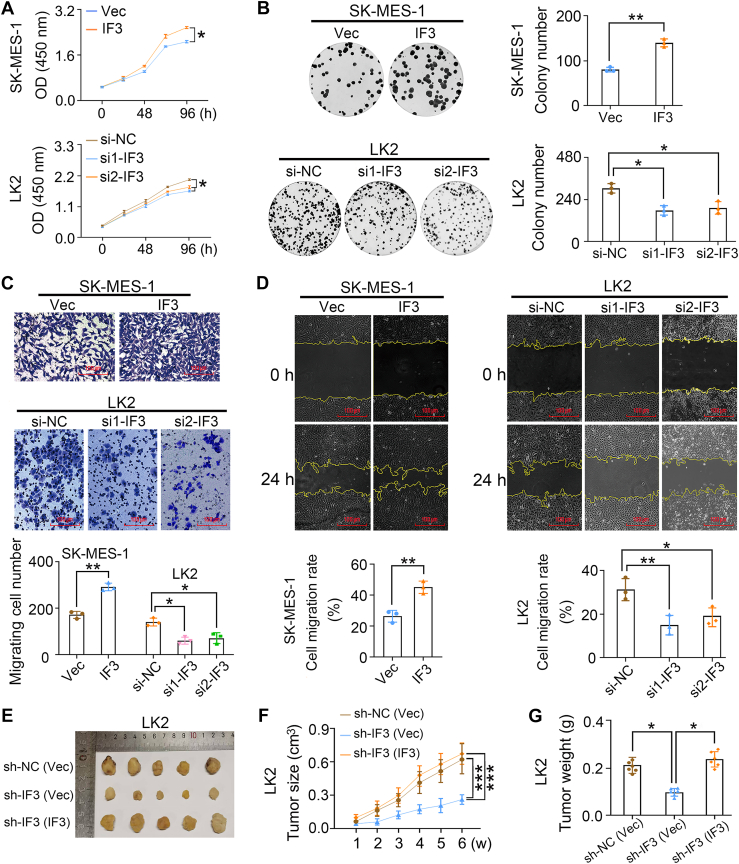
**Effect of IFIT3 on the biological behavior of LUSC and LCLC cells.***A*, viability. *B*, colony formation. *C*, transwell migration. *D*, wound healing. *E and G*, subcutaneously injected tumors (G418 screening, n = 5). *B and D*, the representative images (the scale bar represents 100 μm) and statistical analysis graphs. Vec, vector, the control group of the IFIT3 expression plasmid. IF3, transfection of IFIT3 expression plasmid. si-NC, siRNA-negative control, a scrambled siRNA was selected as a negative control. si1-IF3 and si2-IF3, siRNA1-IFIT3 and siRNA2-IFIT3, two pairs of siRNAs for IFIT3. sh-IF3, shRNA-IFIT3. sh-NC, shRNA-negative control, a scrambled shRNA was selected as a negative control. sh-IF3 (IF3), following IFIT3 knockdown with shRNA, its expression was restored by transfection with IFIT3 expression plasmid. Error bars indicated mean ± SD (n = 3) for (*A*), mean ± SEM (n = 3) for (*B–D*), and mean ± SD (n = 5 for each group) for (*F and G*). Data were analyzed by two-way ANOVA with Šídák’s *post hoc* test for (*A* and *F*), two-tailed unpaired Student’s *t* test for (*B–D*) in SK-MES-1 cell, and one-way ANOVA with Dunnett’s or Tukey’s *post hoc* test for (*B–D*, and *G*) in LK2 cell. ∗*p* < 0.05, ∗∗*p* < 0.01, ∗∗∗*p* < 0.001. LCLC, large-cell lung carcinoma; LUSC, lung squamous cell carcinoma.

To investigate the effects of IFIT3 on the biological functions, H460 (LCLC cell) and SK-MES-1 (LUSC cell) were transfected with IFIT3 expression plasmid, while LK2 (LUSC cell) was transfected with IFIT3 siRNA (Fig. S1), confirming the specificity of the primary antibody and the observed effects. IFIT3 overexpression promoted the viability, colony formation, transwell migration, and wound healing of SK-MES-1 cells (Fig. 2, *A*–*D*, *p* < 0.05). Conversely, IFIT3 knockdown using IFIT3 siRNAs inhibited the viability, colony formation, transwell migration, and wound healing of LK2 cells (Fig. 2, *A*–*D*, *p* < 0.05). Consistent phenotypes with rescue reversal confirm IFIT3-specific effects (Figs. S2A and S3). IFIT3 positively regulated the transwell migration, even under conditions where the proliferation was pharmacologically inhibited by mitomycin C (Fig. S4).

Having established the *in vitro* relevance of IFIT3, we next assessed the tumorigenic effect of IFIT3 on LUSC/LCLC *in vivo*. The volume and weight of subcutaneously injected tumors were lower in the IFIT3 shRNA transfection group than in the control group of LK2 cells, and consistent phenotypes with rescue reversal confirm IFIT3-specific effects (Figs. 2, *E*–*G* and S2*B*, *p* < 0.05).

**Figure 3:**
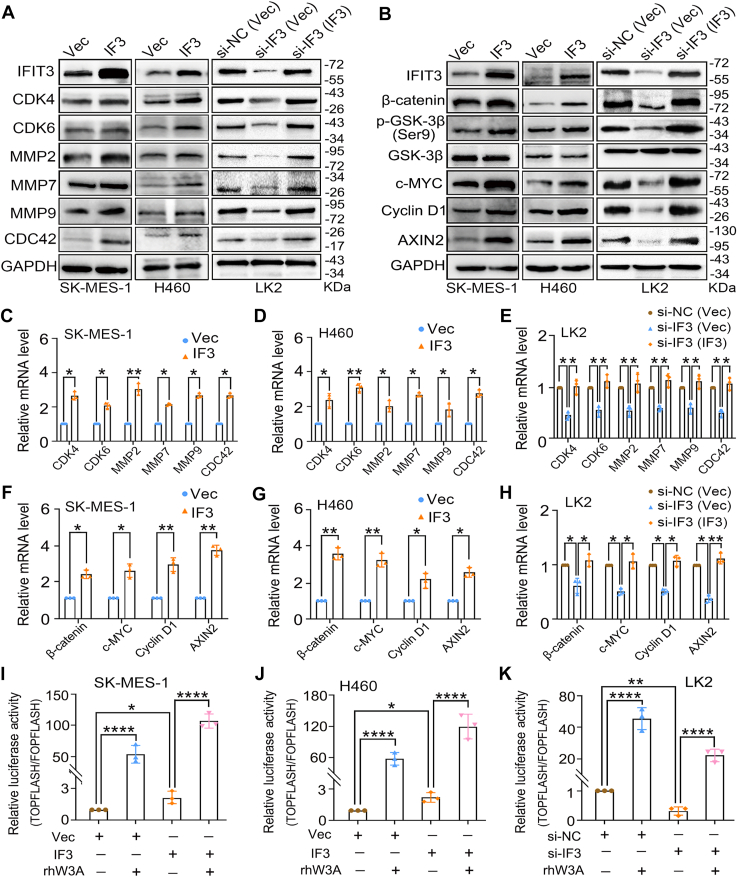
**Effect of IFIT3 on cell malignancy and canonical WNT signaling.***A and B*, Western blot analysis. GAPDH was an internal control. Quantitative analysis of band intensities is provided in Figure S5. p-GSK-3β (Ser9), the serine nine phosphorylation of GSK-3β. *C–H*, quantitative real-time PCR analysis. β-actin was an internal control. Vec, vector, the control group of the IFIT3 expression plasmid. IF3, transfection of IFIT3 expression plasmid. si-IF3, siRNA-IFIT3. si-NC, siRNA-negative control, a scrambled siRNA selected as a negative control. si-IF3 (IF3), following IFIT3 knockdown with siRNA, its expression was restored by transfection with IFIT3 expression plasmid. *I–K*, dual-luciferase assay. IFIT3 expression plasmid or IFIT3 siRNA was transfected to LUSC/LCLC cells, with or without the treatment of recombinant WNT3A protein. (i) Negative Control: transfection of empty plasmid vector or siRNA-negative control. (ii) Positive Control: treatment of recombinant WNT3A protein. (iii) Test Group 1: transfection of IFIT3 expression plasmid or IFIT3 siRNA. (iv) Test Group 2: transfection of IFIT3 expression plasmid or IFIT3 siRNA, with the treatment of recombinant WNT3A protein. rhW3A, the cells were treated with recombinant human WNT3A protein (100 ng/ml) for 12 h. Error bars indicated mean ± SEM (n = 3). Data were analyzed by two-tailed unpaired Student’s *t* test for (*C*, *D*, *F* and *G*), and one-way ANOVA with Tukey’s *post hoc* test for (*E*, *H–K*). ∗*p* < 0.05, ∗∗*p* < 0.01, ∗∗∗∗*p* < 0.0001. LCLC, large-cell lung carcinoma; LUSC, lung squamous cell carcinoma.

Western blot analysis and quantitative real-time PCR analysis confirmed that IFIT3 overexpression upregulated the protein and mRNA expressions of cell cycle-related proteins CDK4 and CDK6, RHO GTPase CDC42, and matrix metalloproteinases MMP2, MMP7, and MMP9. Conversely, IFIT3 knockdown using IFIT3 siRNA downregulated the protein and mRNA expressions of these factors (Figs. 3, *A*, *C*–*E* and S5*A*, *p* < 0.05). And the successful restoration of IFIT3 expression in the rescue groups, effectively reversing the siRNA-induced effects. IFIT3 specifically altered the activity of CDC42, but had no significant effect on the activities of RAC1 or RHOA (Fig. S6). Flow cytometry analysis revealed that IFIT3 overexpression significantly decreases the population in G1 phase and increases the population in S phase, whereas IFIT3 knockdown has the opposite effect (Fig. S7, *A* and *C*, Table S1, *p* < 0.05). Manipulation of IFIT3 consistently altered the levels of Cyclin D1 and S780 phosphorylation of retinoblastoma (Rb) (Figs. 3*B* and S7, *B* and *D*, *p* < 0.05). In summary, IFIT3 acts as a multi-faceted driver of LUSC/LCLC malignancy, potentiating tumorigenic phenotypes both in cell and animal models.

### IFIT3 promotes the activation of canonical WNT signaling in LUSC/LCLC cells

Mechanistically, the result of gene set enrichment analysis revealed that high IFIT3 gene expression group was enriched in WNT/β-catenin pathway (Fig. S8*A*). β-catenin (CTNNB1-encoding protein) is a key transcription factor in WNT/β-catenin pathway. The Gene Expression Profiling Interactive Analysis database was used to analyze the correlation between CTNNB1 and IFIT3 expression. The Spearman correlation analysis showed that the expression of CTNNB1 was positively correlated with that of IFIT3 (Fig. S8*B*).

To determine whether IFIT3 activates the canonical WNT pathway, we examined its effect on the expressions of canonical WNT-target genes. Western blot, quantitative real-time PCR analysis, and dual-luciferase assay confirmed that IFIT3 overexpression had positive effects on TOPFLASH activity and the expressions of Cyclin D1, AXIN2, and c-MYC (Figs. 3, *B*, *F*–*K*, S5*B*, and S9, *p* < 0.05). On the contrary, IFIT3 knockdown using IFIT3 siRNA downregulated the above factors (Figs. 3, *B*, *F*–*K*, S5*B* and S9, *p* < 0.05). And the successful restoration of IFIT3 expression in the rescue groups, effectively reversing the siRNA-induced effects. IFIT3 had no effect on β-catenin-independent noncanonical WNT signaling activity (Fig. S10). WNT3A treatment (100 ng/ml, 12 h) induced a strong increase in TOPFLASH activity. Importantly, IFIT3 synergized with WNT3A treatment, further elevates the activity (Fig. 3, *I*–*K*, *p* < 0.05). IFIT3 overexpression increased the β-catenin expression, while WNT3A synergized with it further elevated the β-catenin expression (Fig. S11, *p* < 0.05), confirming IFIT3's role as a positive regulator of canonical WNT signaling.

**Figure 4:**
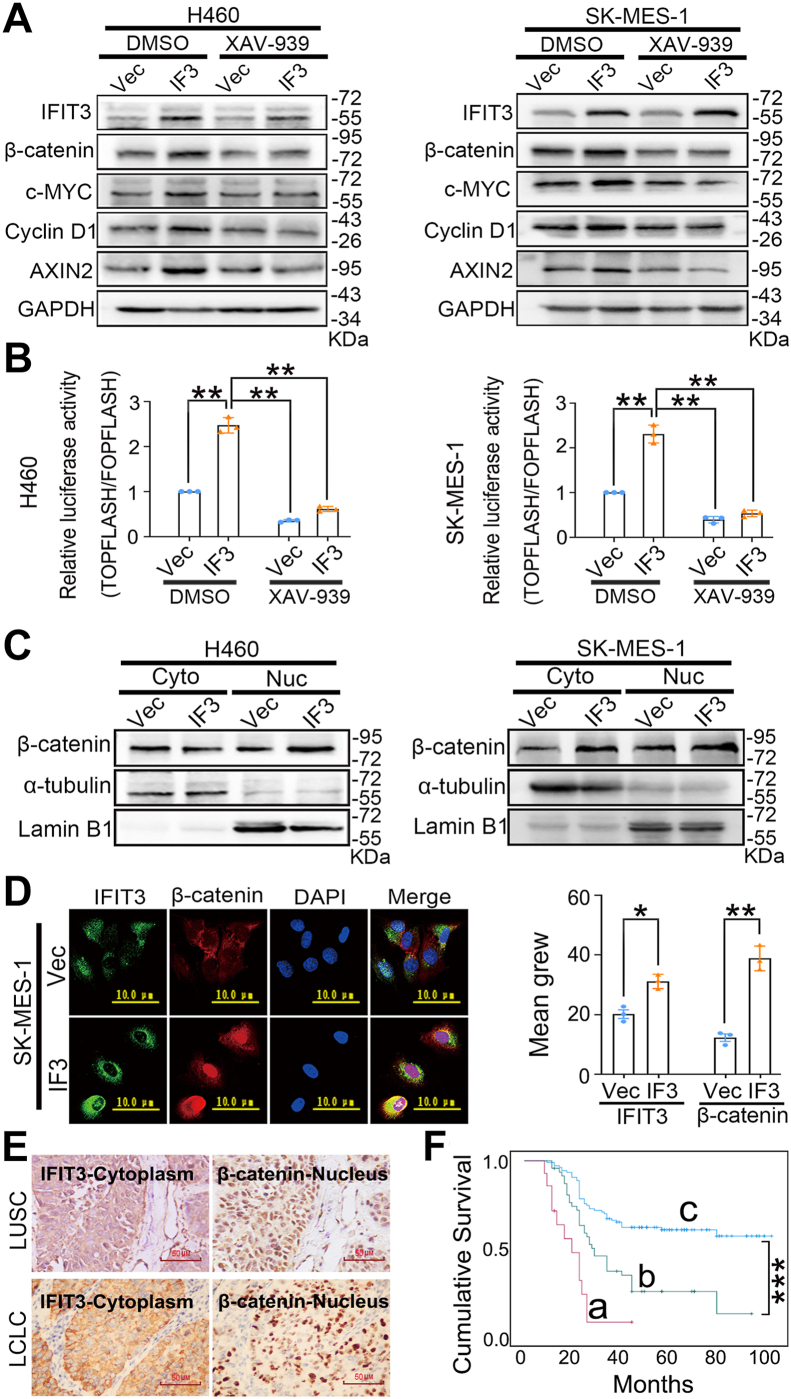
**Effect of IFIT3 on canonical WNT signaling.***A*, Western blot analysis. GAPDH was an internal control. Quantitative analysis of band intensities is provided in Figure S12*A*. Vec, vector, is the control group of the IFIT3 expression plasmid. IF3, transfection of IFIT3 expression plasmid. After transfection with IFIT3 expression plasmid, cells were treated for 24 h with dimethyl sulfoxide (10 μM; IFIT3+ dimethyl sulfoxide group) or XAV-939 (10 μM; IFIT3+XAV-939 group). *B*, dual-luciferase assay. TOPFLASH/FOPFLASH was used to measure for β-catenin/TCF mediated transcriptional activity. *C*, nuclear and Cytoplasmic Protein Extraction Assay analysis. Cyto, cytoplasm; Nuc, nucleus; Lamin B1 was a reference nuclear protein, and α-tubulin was a positive control for the cytoplasm. Quantitative analysis of band intensities is provided in Figure S12*B*. *D*, immunofluorescence analysis (the scale bar represents 10 μm). Quantification of average fluorescence intensity was provided. *E*, representative immunohistochemistry images for IFIT3 and β-catenin in LUSC and LCLC (magnification: × 400; the scale bar represents 50 μm). *F*, Kaplan-Meier survival curve analyzing the different group in cancerous samples from LUSC/LCLC patients. a, the group with cytoplasmic IFIT3 and nuclear β-catenin coexpression (16 cases); b, the group with IFIT3 and β-catenin coexpression in the cytoplasm (56 cases); c. others (110 cases). Log-rank test was used for survival comparison. Error bars indicated mean ± SEM (n = 3). Data were analyzed by one-way ANOVA with Tukey’s *post hoc* test for (*B*), and two-tailed unpaired Student’s *t* test for (*D*). ∗*p* < 0.05, ∗∗*p* < 0.01, ∗∗∗*p* < 0.001. LCLC, large-cell lung carcinoma; LUSC, lung squamous cell carcinoma.

XAV-939 is a tankyrase inhibitor that promotes β-catenin degradation (13). To further investigate the role of IFIT3 in WNT/β-catenin signaling, XAV-939 was used to treat H460 and SK-MES-1 cells transfected with IFIT3 expression plasmid. The upregulated expressions of Cyclin D1, AXIN2, c-MYC, and β-catenin and the strongly activated WNT/β-catenin signaling induced by IFIT3 transfection were significantly inhibited by XAV-939 (Figs. 4, *A* and *B*, and S12*A*, *p* < 0.05).

Given our findings that IFIT3 activates the canonical WNT pathway, we next explore the underlying mechanism. Western blot analysis revealed that IFIT3 positively regulated the expression of β-catenin and the serine 9 (Ser9) phosphorylation of GSK-3β (Figs. 3*B* and S5*B*, *p* < 0.05). Nuclear and cytoplasmic protein extraction and immunofluorescence assays showed that IFIT3 promoted β-catenin nuclear translocation (Figs. 4, *C* and *D*, S12*B*, and S13, *p* < 0.05).

β-catenin expression was assessed in 182 NSCLC samples (including LUSC and LCLC) using immunohistochemical staining. The result revealed that 71.4% of LUSC/LCLC tissue samples (130/182) showed positive β-catenin expression (Table 2). Coexpression of IFIT3 and β-catenin in LUSC/LCLC was significantly correlated (Table 2, *p* = 0.003, r = 0.220). Kaplan-Meier survival analysis showed a significantly lower survival time in patients with coexpression of cytoplasmic IFIT3 and cytoplasmic/nuclear β-catenin than those with no coexpression (Fig. 4, *E* and *F*, *p* < 0.05). And there is a significantly lower survival time in patients with coexpression of cytoplasmic IFIT3 and nuclear β-catenin than those with cytoplasmic coexpression of IFIT3 and β-catenin (Fig. 4, *E* and *F*, *p* < 0.05). Multivariate Cox proportional hazards analysis revealed that the coexpression of cytoplasmic IFIT3 and cytoplasmic/nuclear β-catenin remained an independent significant predictor of poor survival of LUSC/LCLC patients (Table S2). Taken together, IFIT3 exerts its oncogenic effect primarily through activating the WNT/β-catenin pathway.

**Table 2:** The relationship between IFIT3 and β-catenin

β-catenin expression	IFIT3 expression		Total
	Negative	Positive	
Negative	21	31	52
Positive	25	105	130

DVL2, the predominant isoform interacting with IFIT3 in LUSC/LCLC cells, activates canonical WNT signaling more significantly than DVL1 or DVL3 upon this interaction.

**Figure 5:**
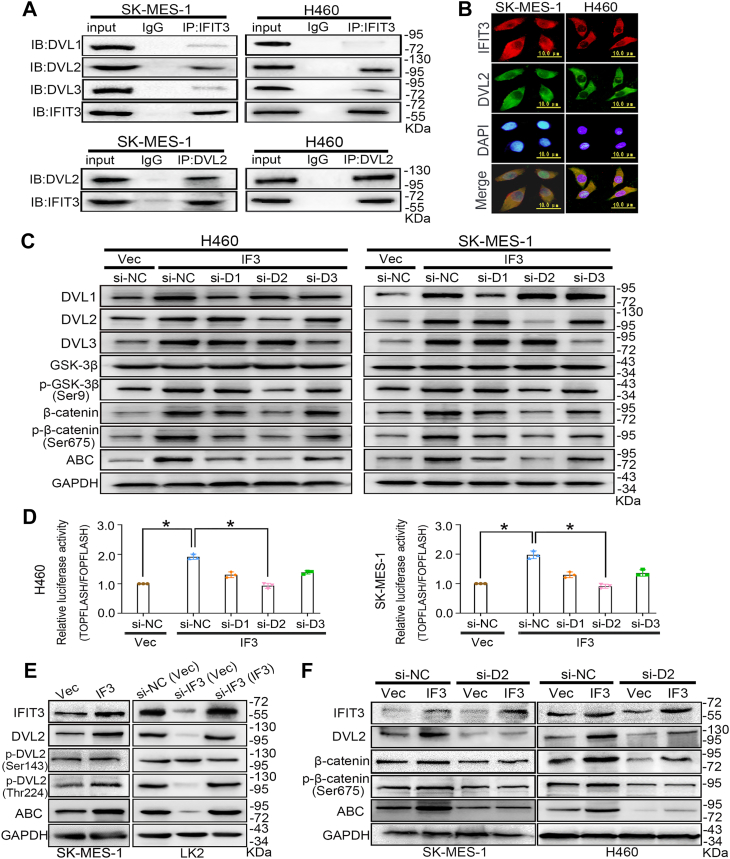
**IFIT3 interacts with DVL2 to promote DVL2 phosphorylation and upregulate β-catenin expression.***A*, co-immunoprecipitation analysis. IP, immunoprecipitation. IB, immunoblot. *B*, immunofluorescence assay (the scale bar represents 50 μm). Quantitative colocalization analysis of these images is presented in Figure S18. *C, E and F*, Western blot analysis. GAPDH was an internal control. Quantitative analysis of band intensities is provided in Figure S14. p-GSK-3β (Ser9), the serine nine phosphorylation of GSK-3β. p-β-catenin (Ser675), the serine 675 phosphorylation of β-catenin. ABC, active non-phospho (Ser33/37/Thr41) β-catenin. p-DVL2 (Ser143), the serine 143 phosphorylation of DVL2. p-DVL2 (Thr224), the threonine 224 phosphorylation of DVL2. *D*, dual-luciferase assay. TOPFLASH/FOPFLASH was measured for β-catenin/TCF mediated transcriptional activity. Cells were co-transfected with siRNA and plasmid. Vec, vector, the control group of the IFIT3 expression plasmid. IF3, transfection of IFIT3 expression plasmid. si-D1, siRNA-DVL1. si-D2, siRNA-DVL2. si-D3, siRNA-DVL3. si-NC, siRNA-negative control, a scrambled siRNA selected as a negative control. si-IF3 (IF3), following IFIT3 knockdown with siRNA, its expression was restored by transfection with IFIT3 expression plasmid. Data were analyzed by one-way ANOVA with Tukey’s *post hoc* test for (*D*). Error bars indicated mean ± SEM (n = 3). ∗*p* < 0.05.

To demonstrate the detailed mechanism of IFIT3 regulating canonical WNT signaling in LUSC and LCLC cells, we detected the relationship between IFIT3 and dishevelled (DVL), a key component of WNT/β-catenin pathway (14). Co-immunoprecipitation assay was applied to test whether IFIT3 interacts with three DVL isoforms. Cells were lysed and immunoprecipitated with IFIT3 antibody. Western blot analysis showed that each DVL isoform has an interaction with IFIT3 (Fig. 5*A*).

To explore the role of three DVL isoforms on IFIT3 regulating canonical WNT pathway, IFIT3 expression plasmid and DVL1/DVL2/DVL3 siRNA were co-transfected into H460 and SK-MES-1 cells. Western blot analysis showed that IFIT3 positively regulated the Ser9 phosphorylation of GSK-3β, the Ser675 phosphorylation of β-catenin, and the β-catenin and active nonphospho (Ser33/37/Thr41) β-catenin expressions; DVL2 siRNA has a stronger inhibitory effect on the upregulating above factors induced by IFIT3 transfection than DVL1 or DVL3 siRNA (Figs. 5*C* and S14, *A* and *B*, *p* < 0.05). Dual-luciferase assay confirmed DVL2 knockdown has a stronger inhibitory effect on activating WNT/β-catenin signaling induced by IFIT3 transfection than that DVL1 or DVL3 knockdown (Fig. 5*D*, *p* < 0.05). And the successful restoration of DVLs expression in the rescue groups, effectively reversing the siRNA-induced above effects (Figs. S15–S17).

To further explore the underlying mechanism by which IFIT3 activates canonical WNT signaling, we assessed the activation of the pathway's effector, DVL2. The immunofluorescence images revealed a significant co-localization between IFIT3 and DVL2 in cytoplasm (Fig. 5*B*). As shown in Figure S18, the IFIT3 red fluorescence signal colocalized well with the DVL2 green fluorescence signal, with the Spearman's correlation coefficient of 0.792 and 0.723. IFIT3 was also immunoprecipitated using the DVL2 antibody in SK-MES-1 and H460 cells (Fig. 5*A*). Dual-luciferase assay showed that DVL2 knockdown inhibited the activation of canonical WNT/β-catenin signaling mediated by IFIT3 (Fig. S19, *p* < 0.05). IFIT3 overexpression significantly enhanced the phosphorylation of DVL2 at threonine 224 (Thr224), the dephosphorylation of β-catenin at Ser33/37/Thr41 (active β-catenin) in SK-MES-1 cells, whereas IFIT3 knockdown significantly inhibited the above expressions in LK2 cells (Figs. 5*E* and S14, *C* and *D*, *p* < 0.05). And the successful restoration of IFIT3 expression in the rescue groups, effectively reversing the siRNA-induced effects. However, the Ser143 phosphorylation of DVL2 had not significant changes in IFIT3-knockdown/overexpression cells (Figs. 5*E* and S14, *C* and *D*, *p* > 0.05), confirming that the Thr224 phosphorylation of DVL2 contributes to the activation of WNT/β-catenin signaling mediated by IFIT3. Also, IFIT3 positively regulated the Ser675 phosphorylation of β-catenin and the β-catenin and active β-catenin expressions; DVL2 siRNA has a significant inhibitory effect on the upregulating the above factors induced by IFIT3 transfection (Figs. 5*F* and S14, *E* and *F*, *p* < 0.05).

**Figure 6:**
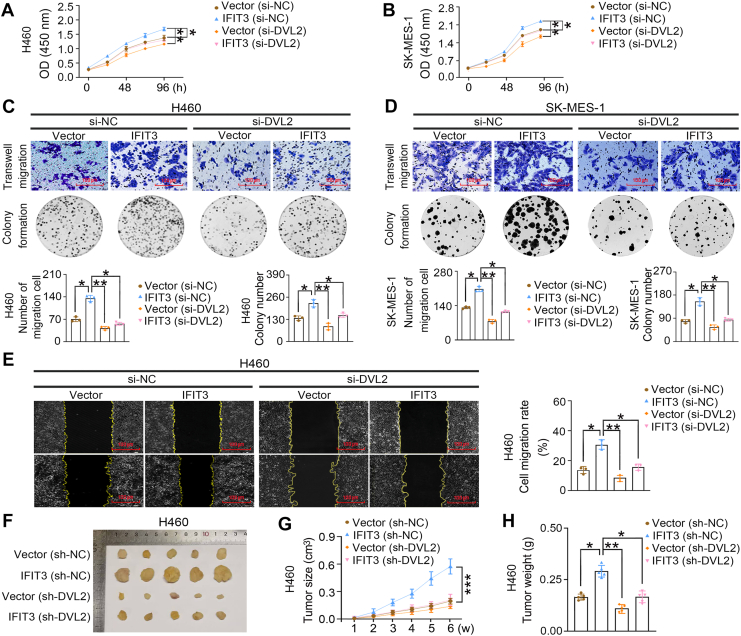
**Effects of IFIT3 and DVL2 on the biological behavior of LUSC and LCLC cells.***A and B*, viability. *C and D*, transwell migration and colony formation. (*E*) Wound healing. *F and H*, subcutaneously injected tumors (G418 screening, n = 5). *C and E*, the representative images (scale bar, 100 μm) and statistical analysis graphs. H460 and SK-MES-1 cells were cotransfected with siRNA/shRNA and plasmid. Vector is the control group of the IFIT3 expression plasmid; IFIT3, transfection of IFIT3 expression plasmid. si-DVL2, siRNA-DVL2. si-NC, siRNA-negative control, a scrambled siRNA was selected as a negative control. sh-DVL2, shRNA-DVL2. sh-NC, shRNA-negative control, a scrambled shRNA was selected as a negative control. Error bars indicated mean ± SD (n = 3) for (*A* and *B*), mean ± SEM (n = 3) for (*C–E*), and mean ± SD (n = 5 for each group) for (*G* and *H*). Data were analyzed by two-way ANOVA with Šídák’s *post hoc* test for (*A*, *B*, and *G*), and one-way ANOVA with Tukey’s *post hoc* test for (*C–E*, and *H*). ∗*p* < 0.05, ∗∗*p* < 0.01, ∗∗∗*p* < 0.001. LCLC, large-cell lung carcinoma; LUSC, lung squamous cell carcinoma.

Finally, to confirm the interaction between IFIT3 and DVL2 is critical, DVL2 overexpression was performed on the background of IFIT3 knockdown. Western blot analysis, dual-luciferase assay and quantitative real-time PCR analysis showed that DVL2 overexpression activated canonical WNT signaling, but this activation was significantly attenuated by IFIT3 knockdown (Figs. S2*C* and S20, *p* < 0.05). DVL2 knockdown also inhibited the malignant phenotypes of H460 and SK-MES-1 cells mediated by IFIT3 *in vitro* and *in vivo* (Fig. 6, *p* < 0.05). And the successful restoration of DVL2 expression effectively reversing the siRNA/shRNA-induced effects (Figs. S2*D*, S21 and S22). DVL2 overexpression promotes the malignant phenotype of LK2 cells *in vitro*, but this promotion was significantly attenuated by IFIT3 knockdown (Fig. S3, *p* < 0.05). Therefore, the IFIT3-DVL2 interaction is pivotal for the enhanced the canonical WNT signaling in LUSC and LCLC.

## Discussion

Malignancy poses a serious threat to human health, with its increasing morbidity and mortality imposing a substantial economic burden on society. Various therapeutic drugs have been developed over the years based on the histopathological and molecular pathological characteristics of malignancies. However, drug resistance and side effects have increased with the increasing frequency of their application. Understanding the pathogenesis of malignancies is crucial for developing effective interventions to improve patient prognosis and manifestations. Malignancy is a complex disease involving a balance between oncogenes and tumor suppressor genes that directly alter the activity of related metabolic enzymes (15). Metabolic abnormalities in malignant cells are closely related to various dysregulated signaling pathways. Multiple target genes and proteins involved in these signaling pathways exhibit abnormal expression. Therefore, exploring targeted protein factors and signaling pathways is important for reducing mortality and improving the outcome of patients with malignancies.

CDK4 and CDK6 are well-established central regulators of the G1/S phase cell cycle transition, and their dysregulation is a hallmark of uncontrolled proliferation in cancer cell. Altered CDK4/6 expression is related to the malignant progression (16, 17). Matrix metalloproteinases (MMPs) are critically involved in degrading extracellular matrix (ECM) components, facilitating cancer metastasis. Elevated MMPs expressions are frequently observed in lung cancer (18, 19). Cell migration is fundamentally driven by dynamic reorganization of the actin cytoskeleton. CDC42, a member of the Rho GTPase family, is a master regulator of actin polymerization. CDC42 activity is frequently dysregulated in lung cancer (20). Importantly, canonical WNT signaling involved in IFIT3 regulating the NSCLC progression and Rho GTPases signaling engage in extensive crosstalk to promote cancer progression (21). Furthermore, other IFIT isoforms often implicated in cancer metastasis (22, 23). The functional assays demonstrate that IFIT3 acts as a positive regulator of malignant phenotypes in LUSC/LCLC cells. Examining CDK4/6, MMP2/7/9 and CDC42 was therefore a logical step to explore potential mediators of IFIT3-driven proliferation and migration. Western blot and quantitative real-time PCR analysis confirmed that IFIT3 positively regulated the expressions of CDK4/6, MMP2/7/9, and CDC42.

IFIT3 play critical roles in malignant progression. In hepatocellular carcinoma, IFIT3 overexpression promotes the malignant progression of tumor cells (8). Among patients with NSCLC, IFIT3 has a bilateral symmetry effect on the malignant progression of LUAD (11, 12). The tumor-suppressive role of IFIT3 in LUAD cell A549 and LUSC cell Calu-1 has been reported (24). The potential reasons for the divergent results might be the followings: (a) Genetic background can vary dramatically across different cancer subtypes. Our SK-MES-1, LK2, and H460 models distinct genetic landscapes (*e.g.*, TP53, PIK3CA-mutation, or EGFR amplification), compared to the A549 and Calu-1 (KRAS-mutation) models. Downstream signaling networks or interacting partners of IFIT3 differ in these contexts, leading to opposing outcomes. (b) The specific constellation of other proteins presents in the cell, and the interaction with different oncogenic drivers could switch IFIT3 from a tumor suppressor to a promoter. (c) Differences in how the cells interact with their microenvironment in experimental culture conditions could also influence the observed phenotype. The conflicting reports within the A549 cell are the evidence for context-dependency (11, 12). Dual nature of IFIT3 may contribute to the heterogeneity of lung cancer and could have implications for patient stratification in the future. Our findings observed high IFIT3 expression in LUSC and LCLC tissue specimens. IFIT3 overexpression was positively correlated with advanced TNM stage and lymph node metastasis in patients with LUSC and LCLC. IFIT3 promotes the malignant phenotypes of LUSC and LCLC cells *in vitro* and *in vivo*. These results indicated that IFIT3 acts as a cancer promoter in LUSC and LCLC.

To understand the pathogenesis, we applied the gene set enrichment analysis and Gene Expression Profiling Interactive Analysis database analyses and found that IFIT3 was positively correlated with the WNT/β-catenin signaling pathway. WNT/β-catenin pathway is a conserved signaling axis involved in various physiological processes such as proliferation, embryogenesis, and organ development (25). β-catenin is the central effector of the canonical WNT pathway. In many cancer types, aberrant activation of this pathway leads to sustained nuclear accumulation of β-catenin. In the nucleus, it partners with T cell-specific factor/lymphoid enhancer-binding factor transcription factors to drive the expression of pro-tumorigenic genes (26, 27). Specifically, dysregulation of canonical WNT pathway is a well-established oncogenic driver in NSCLC (28), which is strongly associated with poor patient survival. In the absence of WNT signal, GSK-3β forms a destruction complex that phosphorylates β-catenin, targeting it for proteasomal degradation (29). GSK-3β phosphorylation at Ser9 site inhibits GSK-3β kinase activity (30). When GSK-3β (Ser9) phosphorylation is increased, β-catenin degradation is suppressed, leading to β-catenin accumulation and activation of the downstream transcriptional program. Our measuring GSK-3β (Ser9) phosphorylation was to directly test whether IFIT3 impacts this critical regulatory node.

With GSK-3β activity inhibited and clinically significant mutations, β-catenin can no longer be phosphorylated at Ser33, Ser37, and Thr41 (31). The non-phosphorylated form of β-catenin is now stable and escapes from degradation, leading to aberrant cytoplasmic accumulation and nuclear translocation observed in numerous malignancies (32). Therefore, β-catenin that is non-phosphorylated at Ser33/37/Thr41 is the active, stabilized form that functions as the primary downstream effector of canonical WNT signal. Antibodies specific for non-phosphorylated active β-catenin (Ser33/37/Thr41) detect the transcriptionally active, GSK-3β-escape form that drives canonical WNT signaling. Above cascade reactions result in β-catenin cytoplasmic accumulation and nuclear translocation. Additionally, phosphorylation of β-catenin at the Ser675 site can contribute to its cytoplasmic stabilization and nuclear accumulation, potentially acting as a supplementary regulatory mechanism alongside the primary N-terminal degron motif (Ser33/Ser37/Thr41) (33). β-catenin interacts with T cell-specific factor/lymphoid enhancer-binding factor in the nucleus, resulting in the upregulation of WNT-target genes and promoting carcinogenesis (34, 35). Bioinformatics analysis suggests a potential correlation between IFIT3 and canonical WNT pathway. Examining the coexpression of IFIT3 and β-catenin in LUSC/LCLC samples would provide the first clinical evidence supporting their functional link in human tumors. β-catenin subcellular localization is a direct functional readout of pathway activity. Cytoplasmic localization generally indicates an inactive state, whereas nuclear translocation is the definitive hallmark of an active canonical WNT signal (36). Our study focused on IFIT3 to elucidate the mechanism underlying its role in WNT/β-catenin signaling. In LUAD and LCLC cells, IFIT3 promoted β-catenin nuclear translocation by enhancing the Ser9 phosphorylation of GSK-3β and Ser675 phosphorylation of β-catenin and positively regulating the target genes of the WNT/β-catenin pathway.

In canonical WNT pathway, phosphorylation of DVL is a pivotal regulatory switch. Upon WNT ligand binding, DVL is recruited to the membrane and undergoes activating phosphorylation (37, 38, 39, 40). This form of DVL recruits the AXIN/APC/GSK-3β destruction complex, thereby inhibiting the phosphorylation-dependent degradation of β-catenin. Consequently, cytosolic accumulation of β-catenin subsequently promotes its nuclear translocation (41). Given our focus on validating the role of the canonical WNT pathway in IFIT3-mediated LUSC/LCLC progression, the analysis of DVL phosphorylation—a master regulator of this pathway—was a logical and critical step. DVL includes three domains: DIX, DEP, and PDZ (42, 43). IFIT3 has multiple TPR motifs, and the interaction between the TPR motif and PDZ domain contributes to the binding of LGN (containing the TPR motif) to FRMPD1 (containing the PDZ domain) (44). The finding that IFIT3 interacts with all three DVL isoforms prompted us to narrow our focus. Based on compelling evidence indicating that DVL2 is the key mediator of IFIT3's effect on canonical WNT pathway activation, we concentrated our efforts on elucidating the phosphorylation status of DVL2.

DVL2 phosphorylation at Thr224 is primarily mediated by casein kinase one epsilon, a kinase directly recruited upon WNT pathway activation (45). The regulatory landscape of Ser143 is more complex and context-dependent. DVL2 phosphorylation at Ser143 might play a role in the switch between the canonical and noncanonical WNT pathways (46, 47). The phosphorylation event is essential for downstream signal transduction. Therefore, Thr224 and Ser143 were used as the primary readout for DVL2 activation status. The investigation of DVL2 phosphorylation site under IFIT3 conditions was sought to determine whether IFIT3's oncogenic functions in LUSC and LCLC are mediated through the specific activation of canonical WNT pathway. The finding that IFIT3 specifically enhances DVL2 phosphorylation at Thr224 provides a compelling mechanistic clue.

Our study has a few limitations. The siRNA-IFIT3 treatment reduced LK2 cell proliferation after 24 h, potentially contributing to a wider gap and reduced migration at this time point. However, IFIT3 transfection in SK-MES-1 cells had no effect on cell proliferation at 24 h. This phenomenon may involve a series of signaling factors, such as epithelial-mesenchymal transition (EMT) and CDKs. CDKs are important for cancer cell proliferation (48). Tumor metastasis is closely related to EMT (49). We plan to explore these mechanisms in future studies.

Dynamic actin reorganization contributes to cell migration (50). The small RHO GTPase CDC42 is involved in the regulation of actin cytoskeleton remodeling. CDC42 governs metastasis, but do not affect cell proliferation (51). Conceptually, distinguishing the effect of proliferative input through WNT/β-catenin signaling and the related mechanisms that feed into invasiveness, which probably involve remodeling the actin cytoskeleton, is important.

Our findings demonstrate that IFIT3 directly interacts with DVL2 and promotes its phosphorylation. While the precise mechanism warrants further investigation, we propose several plausible scenarios. It is possible that IFIT3 acts as a scaffold protein. By binding to DVL2, IFIT3 may recruit and juxtapose kinases responsible for DVL2 phosphorylation, such as CK1δ/ε (52). Alternatively, the interaction with IFIT3 might induce a conformational change in DVL2, rendering its phosphorylation sites more accessible to constitutively active kinases. Furthermore, given the critical role of DVL2 phosphorylation in its polymerization and formation of signalosomes, IFIT3 could potentially stabilize these active polymers, preventing their dephosphorylation by phosphatases and ensuring sustained WNT/β-catenin signaling output. Future studies will include mapping the precise binding domains to understand the structural basis of the interaction, identifying other proteins in the IFIT3-DVL2 complex through co-immunoprecipitation coupled with mass spectrometry, and directly testing the effect of IFIT3 on the kinase activity of CK1δ/ε towards DVL2 *in vitro*. Determining whether IFIT3 influences the subcellular localization of DVL2, particularly its recruitment to the plasma membrane, will also be a critical next step.

Notably, this study revealed high IFIT3 protein expression in LUSC tumor tissues *via* immunohistochemistry, which was associated with poor prognosis. However, analysis of the TCGA database indicated a downregulating trend of IFIT3 mRNA in both LUSC and LUAD tumor tissues (Fig. S23). This inconsistency between transcriptional and protein levels suggests that crucial post-transcriptional regulatory mechanisms may be at play in LUSC (53). For instance, in the context of LUSC/LCLC, IFIT3 protein might stably accumulate through enhanced translational efficiency or by evading protein degradation pathways, thereby exerting its pro-tumorigenic function. This subtype-specific protein regulation pattern, together with the potentially distinct regulatory mechanisms for IFIT3 in LUAD cell line A549 (11, 12), constitutes a significant aspect of lung cancer heterogeneity. Future studies should identify the molecular basis for IFIT3’s divergent roles in lung adenocarcinoma. Our functional experiments directly confirmed the oncogenic role of high IFIT3 protein in LUSC/LCLC, underscoring the importance of investigating its function at the protein level.

Overall, IFIT3 overexpression in LUSC and LCLC was significantly correlated with a poor prognosis. Mechanistic studies demonstrated that DVL2, among the three DVL isoforms, most critically interacts with IFIT3. The IFIT3-DVL2 interaction enhances the phosphorylation of DVL2 to enhance β-catenin nuclear translocation, which activates canonical WNT signaling and leads to the progression of LUSC and LCLC. This pathway may provide novel insights into targeted therapy for patients with lung cancer.

## Experimental procedures

### Patients and specimens

We collected 182 resected NSCLC tissue specimens and two resected spleen tissue specimens from patients with an average age of 60 years, ranging from 18 to 77 years. From 2013 to 2015, the patients underwent surgery at the First Hospital of China Medical University, and none received radiotherapy or chemotherapy before resection. All patients or their family members provided informed consent according to the Helsinki Declaration on human research ethics. The study procedure was approved by the Institutional Review Board of the First Hospital of China Medical University in accordance with the Declaration of Helsinki (No. 2025506). Tumor specimens were obtained during surgical resection. The histological subtype was based on the 2017 classification criteria for lung cancer by the World Health Organization, and the TNM stage was based on the International Association for the Study of Lung Cancer Staging Project. Complete follow-up data were available for all the patients. The clinicopathological characteristics of all the patients are presented in Table 1.

### Cell culture

Human bronchial epithelial cells were obtained from the American Type Culture Collection. NSCLC cell lines (A549, LK2, SK-MES-1, H460, and H1299) and the breast cancer cell line MDA-MB-231 were obtained from the Shanghai Cell Bank. The cell lines were authenticated using short tandem repeat DNA profiling and cultured according to the manufacturer’s instructions.

### Reagents

Human pCDNA3.1 3 × Flag tagged IFIT3 was purchased from MIAOLING BIOLOGY. Small interfering RNAs (siRNAs) against IFIT3, DVL1, and DVL2 were designed by GenePharma (details are presented in) Table S3. Two independent siRNAs against IFIT3 were used for functional validation. shRNAs against IFIT3 and DVL2 were purchased from Genechem. siRNA against DVL3 (SR301307) was purchased from Origene. Cells were transfected with the above-mentioned plasmids or siRNAs using Lipofectamine 2000 (Thermo Fisher Scientific). For the rescue experiments, cells were first transfected with target-specific siRNA/shRNA to achieve knockdown, followed by transfection with an overexpression plasmid encoding the gene to confirm phenotype specificity.

XAV-939 (a tankyrase inhibitor) was purchased from MCE. G418 (Sangon, Shanghai, China) at 200 μg/ml was used for screening cells to obtain stable, expressing cell lines. Mitomycin C was purchased from Enzo Life Sciences (Inc). For analysis of WNT3A response, cells were treated with recombinant human WNT3A protein (100 ng/ml, R&D systems, Cat# 5036-WN-500/CF) for 12 h prior to dual-luciferase assay or Western blot analysis.

### Immunohistochemistry (IHC)

Surgically removed NSCLC specimens were fixed in 10% neutral formalin, dehydrated, and embedded in paraffin. Four micrometre -thick sections were obtained through manual slicing. The slides were deparaffinized in xylene, rehydrated with graded alcohol, and boiled in citrate buffer (0.01 M, pH 6.0) for 30 min in a water bath. After cooling at room temperature (22–25 °C), 0.3% hydrogen peroxide was used to inhibit endogenous peroxide activity. An Elivision super HRP immunohistochemistry (IHC) Kit (Maixin Biotechnology) was used for immunohistochemical staining according to the manufacturer’s instructions. Each tissue section was incubated with primary antibodies overnight at 4 °C in a humid chamber, followed by incubation with biotin-labeled secondary antibodies. The sections were counterstained with hematoxylin and dehydrated with ethanol before mounting. The primary antibodies used are listed in Table S4. Rabbit IgG was used as the isotype control instead of the primary antibody (Fig. S24).

The IFIT3-staining intensity was scored as follows: 0 (no staining); 1 (weak staining); 2 (moderate staining); and 3 (strong staining). Scores for IFIT3 staining were assigned as follows: 1 (1–25%), 2 (26–50%), 3 (51–75%), and 4 (76–100%). Both scores were multiplied to obtain a final score of 0 to 12. Scores of ≥4 indicated IFIT3 overexpression (referred to as positive expression), and scores of <4 indicated weak expression (referred to as negative expression).

Two pathologists independently evaluated IHC result using the histo-score (H-score) method, which combines staining intensity (0–3) and percentage of positive cells (0–100%). This analysis was performed in 5 × 400 fields (excluding necrosis) with interobserver concordance. The H-score had an analytical range of 0 to 300 and was calculated using the following formula: H-score=[0×(%negative)+1×(%weak+)+2×(%moderate+)+3×(%strong+)].

### Western blot analysis

Cells were plated in each well of a 6-well culture plate at 2 × 10^5^–5 × 10^5^ cells 1 day before transfection. After 24 h of transfection, the cells were collected and lysed (RIPA: PMSF: NAF: NA_3_VO_3_ = 100: 1: 1: 1). The lysates were centrifuged at 12,000 rpm at 4 °C for 20 min, and the protein concentration in the supernatant was detected by using a bicinchoninic acid assay. Supernatants were separated by SDS-PAGE and transferred onto polyvinylidene difluoride membranes (Beyotime Institute of Biotechnology). After transferring, the membranes were blocked in 5% skim milk or 5% bovine serum albumin (BSA) at room temperature (22–25 °C) for 2 h, followed by incubation with the indicated primary antibodies overnight at 4 °C. The next day, the membranes were incubated with horseradish peroxidase-conjugated secondary antibodies at room temperature for 2 h. Finally, protein bands on the membranes were detected using an enhanced chemiluminescence kit (Thermo Fisher Scientific). A GAPDH antibody was used to control protein loading. The protein bands were analyzed using the ImageJ software (https://imagej.net/ij/index.html, version 1.53a, National Institute of Health). The grayscale values of the targeted bands were normalized to the represent reference band to determine the expression level of the target protein. The results were analyzed using GraphPad Prism 8.0 (https://www.graphpad.com/scientific-software/prism/).

Nuclear and cytosolic proteins were separated using nuclear and cytoplasmic protein extraction kits (Beyotime Institute of Biotechnology), according to the manufacturer’s instructions. Detailed information on the primary antibodies used is provided in Table S4.

### RNA extraction and quantitative real-time PCR analysis

The miRNeasy Mini Kit (Qiagen) was used to extract total RNA from the cells or tissues. The PrimeScript RT Reagent Kit was used for the RNA reverse transcription of total RNA (500 ng). Power SYBR green PCR Master Mix was used for the double-stranded cDNA amplified through quantitative real-time PCR. The expression level of target mRNA was normalized to β-actin. The 2−ΔΔCT method was used to evaluate the relative expression. The sequences of primers used in this study are listed in Table S5.

### Co-immunoprecipitation assay

Cells were cultured in 10-cm dishes. When the cells grew to a fusion degree of 90 to 100%, they were collected and lysed (NP40: PMSF = 100: 1) on ice for 25 min. The supernatant was obtained after centrifugation at 12,000 rpm at 4 °C for 20 min. Each sample was added with approximately 1 μg common IgG and 20 μl of fully resuspended Protein A + G Agarose (Thermo Fisher Scientific), followed by incubation on a shaker at 4 °C for 2 h to block nonspecific binding. The supernatant was then centrifuged at 2500 rpm at 4 °C for 5 min, divided into input, negative control (IgG), and IP groups, and incubated overnight at 4 °C on a shaker. The next day, 40 μl of Protein A + G Agarose was added to the negative control and IP groups and incubated for 3 h at 4 °C on a shaker. After centrifugation at 2500 rpm for 5 min at 4 °C, the supernatant was discarded, and the beads were washed with 500 μl of NP40 four times. A 2 × loading buffer (equal to the volume of the beads) was added, and the samples were boiled for 10 min. The subsequent procedures were described in the same way as for Western blot analysis.

### 3-(4,5-Dimethylthiazol-2-yl)-2,5-diphenyltetrazoliumbromide and colony formation assays

At 24 to 36 h after transfection, the cell suspensions were prepared. Individual wells of 96-well culture plates were seeded with 3 × 10^4^ cells. The inoculated 96-well plates were cultured at the appropriate cell density. Next, the 3-(4,5-dimethylthiazol-2-yl)-2,5-diphenyltetrazoliumbromide (MTT) solution (10 μl, 5 mg/ml, 0.5% MTT) was added into each well and cultured for 4 to 6 h. After discarding the supernatant, 150 μl of dimethyl sulfoxide was added to each well, and the crystal was dissolved by low-speed shaking for 10 min. The absorbance of each well was measured at 450 nm using a microplate reader.

At 24 to 36 h after transfection, the cell suspensions were prepared and seeded into 6-well culture plates at a density of 800 cells per well. Generally, the plate was placed in an incubator (37 °C, 5% CO2) for 7 to 10 days, and the medium was renewed every 5 days. At the end of the culture period, the forming cell colonies were fixed with methanol and stained with 1% crystal violet for 15 min at room temperature (22–25 °C). The stained cell colonies were photographed and counted using Photoshop and ImageJ (1.8.0) and analyzed by GraphPad Prism 8.0, and the experimenter was blinded to group identities during counting. Colonies were counted if they met all the following objective criteria: 1) a minimum diameter of 0.5 mm; 2) a compact, rounded morphology with clear boundaries; 3) being macroscopically visible, irrespective of staining intensity.

### Transwell assay

Transwell membranes (8 μm pore polycarbonate membrane, in 24-well plates) were coated with Matrigel (1 μg/ml, BD Bioscience) on the upper surface of the membrane in every well. In the upper chamber, we cultured the cells (3 × 10^5^ cells) in 100 μl serum-free medium; in the lower chamber, we added the medium containing 10% fetal bovine serum. After incubation at 37 °C with 5% CO_2_ for 24 h, cells diffusing into the membrane were fixed with methyl alcohol and stained with hematoxylin (Sigma). Stained cells were counted under a microscope (Olympus Corporation). Ten fields (400 × magnification) for each filter were randomly selected under a microscope to determine the number of transwell migration cells. The entire field of view was analyzed and counted manually, and the experimenter was blinded to group identities during counting.

### Wound healing assay

Cells were seeded into 6-well culture plates at a density of 5 × 10^5^ cells/well. When the cells reached 100% confluence, scratch wounds were gently created in the center of each well using a 100 μl sterile pipette tip. Cell migration was photographed under an inverted microscope (Olympus Corporation) at 0 h and 24 h after scratching. Cell migration rates were caculated by the following formula: Cellmigrationrate=[(S3+S4)-(S1+S2)]/(S1+S2)%; "S1+S2" represents the area on both sides of the scratch wound measured at 0 h, while "S3+S4" represents the area on both sides of the scratch wound measured at 24 h. Cell migration rates were quantified using GraphPad Prism 8.0. The assay was performed in triplicate.

### Immunofluorescence analysis *via* confocal imaging

Cell suspensions were placed in 24-well culture plates and incubated in Dulbecco’s Modified Eagle Medium or Roswell Park Memorial Institute-1640 medium containing 10% fetal bovine serum. After culturing, cells were fixed with 4% paraformaldehyde at room temperature (22–25 °C) for 20 min and permeabilized with 0.2% Triton X-100 at room temperature for 15 min. The cells were then incubated with 3% BSA for 30 min at room temperature. Then the cells were incubated at 4 °C overnight with a combination of primary antibodies: rabbit anti-IFIT3 antibody together with mouse anti-DVL2 antibody; rabbit anti-IFIT3 antibody together with mouse anti-β-catenin antibody. Detailed information on the primary antibodies used is presented in Table S4. The next day, goat anti-mouse IgG H&L (Alexa Fluor 488) and goat anti-rabbit IgG H&L (Alexa Fluor 594) were added for 1 h at room temperature, and 4′,6-diamidino-2-phenylindole (Thermo Fisher Scientific) was added for 5 min in the dark at room temperature. An FV3000 confocal laser scanning microscope was used to assess protein-protein colocalization. In the FV3000 system, the confocal aperture of the PMT setting tool window is called the confocal pinhole. We set up the control group, the treatment group and each channel in the group to adopt the same confocal aperture value (133 μm). As a positive control, the MDA-MB-231 cell line was used to validate the DVL2 antibody specificity (Fig. S25*A*). The reduced immunoreactivity in cells treated with DVL2 siRNA confirms anti-DVL2 antibody selectivity (Fig. S25*B*). Fluorescence intensity of slices was analyzed by ImageJ software. In fluorescent red images, replace red with magenta. Co-localization claims were supported by pixel-by-pixel correlation (Spearman's rank correlation value).

### Transplanting tumor cells into nude mice

BALB/c mice were treated according to the experimental animal ethics guidelines established by the China Medical University. All animal experiments were approved by the Institutional Animal Research Committee of China Medical University. We purchased specific pathogen-free BALB/c nude mice (4-week-old female; 17.54 ± 0.68 g) from Charles River Laboratories. All experimental procedures involving animals were performed in accordance with the Guidelines for the Care and Use of Laboratory Animals (NIH publications Nos. 80–23, revised 1996). We housed the mice in a controlled room with free access to food and water with a 12-h:12-h light-dark cycle, temperature maintained at 24 °C ± 2 °C, and relative humidity of 55% ± 10%. Wood shavings were added to the cages as bedding material. Before surgical treatment, the mice were anesthetized *via* intraperitoneal injection of pentobarbital sodium (40 mg/kg; Sigma-Aldrich). All experimental procedures were conducted in an specific pathogen-free laboratory during the light phase.

NSCLC cells (H460 cells transfected with IFIT3 expression plasmid and/or a plasmid encoding DVL2 shRNA, LK2 cells transfected with a plasmid encoding IFIT3 shRNA, and the corresponding vector-transfected control cells) were suspended in 0.2 ml of phosphate-buffered saline and subcutaneously inoculated into the right flank (5 × 10^6^ cells per mouse). The following formula was used to calculate the tumor volume: length × width^2^ × 0.5. The animals were randomly assigned to the different treatment groups (n = 5 mice/group). After inoculation for 6 weeks, the nude mice were euthanized by cervical dislocation and then autopsied to collect the tumors.

### Statistical analysis

SPSS (http://www.spss.com) software version 17.0 was used for all the statistical analyses. The chi-square test was used to determine the significance of differences in clinicopathological data. Kaplan–Meier analysis was used to evaluate survival time among the groups of patients and the log-rank test was used to compare the different groups of patients. Independent prognostic factors for overall survival in patients were identified through univariate and multivariate Cox regression analyses. The data is presented as the mean ± SD or mean ± standard error of the mean. Visual statistical graphs were generated using GraphPad Prism version 8.0. Student’s *t* test (with 95% CI) and ANOVA (with 95% CI) were applied to determine statistical significance. Two-tailed Student’s *t* test was used for single comparison (two groups) and one-way or two-way ANOVA with a *post hoc* test was used for multiple comparison (more than two groups). Mann–Whitney U test was applied to compare the H-score from IHC-stained slides between different subgroups. Prior to statistical comparisons, all quantitative data sets were examined for statistical outliers using Grubbs' test or the Tukey's fences method. *p* values < 0.05 were considered to indicate statistical significance (*p* values: ∗*p* < 0.05, ∗∗*p* < 0.01, ∗∗∗*p* < 0.001, and ∗∗∗∗*p* < 0.0001).

### Additional data

This article includes supporting information. Additional information regarding the experimental procedures is provided in the supporting information.

## Data availability

All data pertaining to this study are fully documented within the article and its supporting information. Data can be made available upon request from the lead contact.

## Supporting information

This article contains supporting information.

## Conflict of interest

The authors declare that they have no conflicts of interest regarding the content of this article.
