# Supplementary material for: IFIT3-DVL interaction promotes malignant progression of lung squamous cell carcinoma and large-cell lung carcinoma via canonical WNT signaling
Source: J Biol Chem. 2026 Mar 16;302(5):111368. doi: 10.1016/j.jbc.2026.111368 (PMC13089166; doi:10.1016/j.jbc.2026.111368)
Supplement: Supporting Information [file mmc1.pdf]

IFIT3-DVL interaction promotes malignant progression of lung squamous cell carcinoma and large-cell lung carcinoma via canonical WNT signaling

Authors

Yudie Lu[1], Mengdi Yang[1], Jingrong Zheng[1], Di Zhang[1], Qiang Han[1], Xinran Zhao[1], Minjia Li[2], Ruoqi Zhao[3], Guangping Wu[1], Enhua Wang[1], Huanyu Zhao[1]

Affiliations

[1]Department of Pathology, The First Hospital and College of Basic Medical Sciences, China Medical University, Shenyang, Liaoning, P.R. China

[2]The 109th Class, Basic Medicine, China Medical University, Shenyang, Liaoning, P.R. China

[3]The 110th Class, 8-Year Program, China Medical University, Shenyang, Liaoning, P.R. China.

Correspondence

Huanyu Zhao, Department of Pathology, The First Hospital and College of Basic Medical Sciences, China Medical University; No. 155 Nanjing North Street, Heping District, Shenyang 110001, Liaoning, P.R. China. Telephone/Fax number: 86-24-23261638; Email: zhaohy@cmu.edu.cn

Supplementary Tables

Table S1. Effect of IFIT3 on cell cycle distribution in H460 and LK2 cells.

Group	G1 phase	S phase	G2/M phase
	Mean ± SD (%)	Mean ± SD (%)	Mean ± SD (%)
H460: Vector	77.72 ± 7.07	12.22 ± 1.31	10.06 ± 0.97
H460: IFIT3	62.28 ± 6.32	27.18 ± 2.49	10.54 ± 1.06
LK2: si-NC (Vector)	50.09 ± 3.86	38.58 ± 2.49	11.33 ± 1.75
LK2: si-IFIT3 (Vector)	67.79 ± 6.56	20.12 ± 2.83	12.09 ± 1.49
LK2: si-IFIT3 (IFIT3)	46.72 ± 4.19	42.36 ± 2.92	10.92 ± 1.32

Vector, the control group of IFIT3 expression plasmid.

IFIT3, transfection of IFIT3 expression plasmid.

si-NC, siRNA-negative control, a scrambled siRNA was selected as a negative control.

si-IFIT3, siRNA-IFIT3.

si-IFIT3 (IFIT3), following IFIT3 knockdown with siRNA, its expression was restored by transfection with the IFIT3 expression plasmid.

Table S2. Univariate and multivariate Cox regression analysis for over survival in patients

Variables	Univariate			Multivariate		
	HR	95% CI	P	HR	95% CI	P
Age (years)	1.238	0.788-1.944	0.354			
Gender	1.081	0.681-1.717	0.740			
Histology	1.313	0.615-2.802	0.481			
TNM stage	2.004	1.210-3.318	0.007	2.806	1.801-4.372	<0.001
Lymphatic metastasis	2.031	1.213-3.400	0.007	3.239	1.997-5.253	<0.001
Differentiation	1.110	0.711-1.734	0.646			
IFIT3 expression	2.454	1.222-4.927	0.012	4.616	2.391-8.911	<0.001
β-catenin nuclear expression	2.490	1.568-3.954	<0.001	3.632	2.429-5.433	<0.001
Cytoplasmic IFIT3 and cytoplasmic β-catenin coexpression	1.312	1.014-1.655	0.022	1.530	1.241-1.886	<0.001
Cytoplasmic IFIT3 and nuclear β-catenin coexpression	1.629	1.319-2.011	<0.001	1.699	1.414-2.042	<0.001

Table S3. siRNAs used in this study

siRNA	Sequences
IFIT3 #1	Forward: GAUGUACCAUCUGGAUAAUTT
	Reverse: AUUAUCCAGAUGGUACAUCTT
IFIT3 #2	Forward: GGGACUGAAUCCUCUGAAUTT
	Reverse: AUUCAGAGGAUUCAGUCCCTT
IFIT3 #3	Forward: CACCAAAUUAUUGGUAUCUTT
	Reverse: AGAUACCAAUAAUUUGGUGTT
DVL1	Forward: CCGAGAUGGAAUGGACAAUTT
	Reverse: AUUGUCCAUUCCAUCUCGTT
DVL2	Forward: GUGAGAGCUACCUAGUCAATT
	Reverse: CGCUAAACAUGGAGAAGUATT
Negative Control	Forward: UUCUCCGAACGUGUCACGUTT
	Reverse: ACGUGACACGUUCGGAGAATT

Above siRNAs were designed by GenePharma (Shanghai, China).

DVL3 siRNA was purchased from Origene (Cat# SR301307).

Table S4. Antibodies used in the study.

Antibody	Catalog number &Vendor	Uses
α-tubulin	WL02296 (Wanleibio)	Western blot (1:500)
β-catenin	610153 (Biosciences)	Western blot (1:2000)
		Immunofluorescence (1:100)
Active β-catenin	4270 (CST[#])	Western blot (1:1000)
AXIN2	WLH4725 (Wanleibio)	Western blot (1:500)
CDC42	07-1466 (Sigma- Aldrich)	Western blot (1:500)
CDC42	YT3954 (Immunoway)	Western blot (1:1000)
CDK4	12790 (CST[#])	Western blot (1:1000)
CDK6	13331 (CST[#])	Western blot (1:1000)
C-MYC	67447-1-Ig (Proteintech)	Western blot (1:5000)
Cyclin D1	60186-1-Ig (Proteintech)	Western blot (1:5000)
DVL1	27384-1-AP (Proteintech)	Western blot (1:2000)
DVL2	12037-1-AP (Proteintech)	Western blot (1:2000)
DVL2	sc-8026 (Santa Cruz)	Immunofluorescence (1:50)
		IP (2 μg/IP)
DVL3	13444-1-AP (Proteintech)	Western blot (1:2000)
FLAG M2-peroxidase	A8592 (Sigma-Aldrich)	Western blot (1:500)
GAPDH	60004-1-Ig (Proteintech)	Western blot (1:6000)
GSK-3β	WL01456 (Wanleibio)	Western blot (1:500)
IFIT3	15201-1-AP (Proteintech)	Western blot (1:4000)
		Immunohistochemistry (1:100)
		IP (2 μg/IP)
IFIT3	ab236243 (Abcam)	Immunofluorescence (1:100)
IgG	ab313801 (Abcam)	Immunohistochemistry (1:100)
Lamin B1	WL01775 (Wanleibio)	Western blot (1:500)
MMP2	sc-13595 (Santa Cruz)	Western blot (1:1000)
MMP7	WL04679 (Wanleibio)	Western blot (1:500)

MMP9	sc-21733 (Santa Cruz)	Western blot (1:1000)
P-DVL2 (Ser143)	ab124933 (Abcam)	Western blot (1:2000)
P-DVL2 (Thr224)	ab124941 (Abcam)	Western blot (1:2000)
P-GSK-3β (Ser9)	5558 (CST#)	Western blot (1:1000)
P-Rb	ab47763 (Abcam)	Western blot (1:500)
P-β-catenin (Ser675)	4176 (CST#)	Western blot (1:1000)
RAC1	05-389 (Sigma-Aldrich)	Western blot (1:500)
Rb	sc-102 (Santa Cruz)	Western blot (1:500)
RHOA	AF2179 (Beyotime)	Western blot (1:1000)
WNT3A	2721 (CST#)	Western blot (1:2000)

#CST, Cell Signaling Technology.

Antibody dilution is indicated in bracket.

IP, immunoprecipitation

Table S5. Primer sequences used in this study for quantitative real-time PCR analysis

Gene	Sequences (5'-3')
β-actin	Forward: CTCACCATGGATGATGATATCGC
	Reverse: CACATAGGAATCCTTCTGACCCA
β-catenin	Forward: TAGAAACAGCTCGTTGTACCGCTGGGACCT
	Reverse: GCACTGCCATTTTAGCTCCTTCTTGATGTAAT
AXIN2	Forward: CAGCGAGTATTACTGCTACTCGAAA
	Reverse: TTTTTTGTGCTTTGGGCACTATG
CDC42	Forward: AGGTGTGTGCTGCTATGAAC
	Reverse: CAGGGCATTTGTCATTATTG
CDK4	Forward: GGTGACAAGTGGTGGAACAG
	Reverse: GCCCAATCAGGTCAAAGATT
CDK6	Forward: CGAATGCGTGGCGGAGATC
	Reverse: CCACTGAGGTTAGAGCCATC
Cyclin D1	Forward: GCTGCGAAGTGGAAACCATC
	Reverse: CCTCCTTCTGCACACATTTGAA
c-MYC	Forward: GCGACTCTGAGGAGGAA
	Reverse: TGCGTAGTTGTGCTGATG
IFIT3	Forward: GAAACAGCCATCATGAGTGAGG
	Reverse: TCTTCTAGATCCCTTGAGACACT
MMP2	Forward: AAGGATGGCAAGTACGGCTT
	Reverse: CGCTGGTACAGCTCTCATACTT
MMP7	Forward: GTGGTCACCTACAGGATCGTA
	Reverse: CTGAAGTTTCTATTTCTTTCTTGA
MMP9	Forward: GTACTCGACCTGTACCAGCG
	Reverse: AGAAGCCCCACTTCTTGTCG

These primers were designed by GenePharma (Shanghai, China).

Supplementary Figures

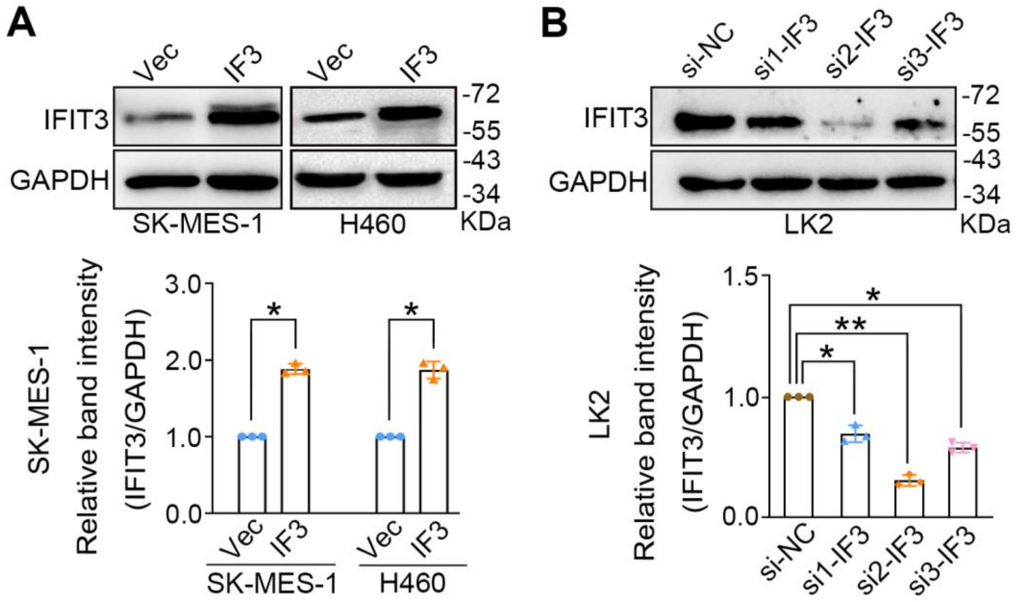

Fig. S1. **IFIT3 expression in LUSC and LCLC cells. (A–B)** Transfection efficiency. Vec, vector, the control group of IFIT3 expression plasmid. IF3, transfection of IFIT3 expression plasmid. si1-IF3, si2-IF3, and si3-IF3, three pairs of siRNAs for IFIT3. si-NC, siRNA-negative control, a scrambled siRNA was selected as a negative control. Two siRNAs demonstrating relatively high knockdown efficiency were chosen for follow-up experiments. Band intensity of IFIT3 was quantified and normalized against that of GAPDH. Data were analyzed by two-tailed unpaired Student's t-test for (A), and one-way ANOVA with Dunnett's post hoc test for (B). Error bars indicated mean ± SEM (n = 3). *p <0.05, **p <0.01.

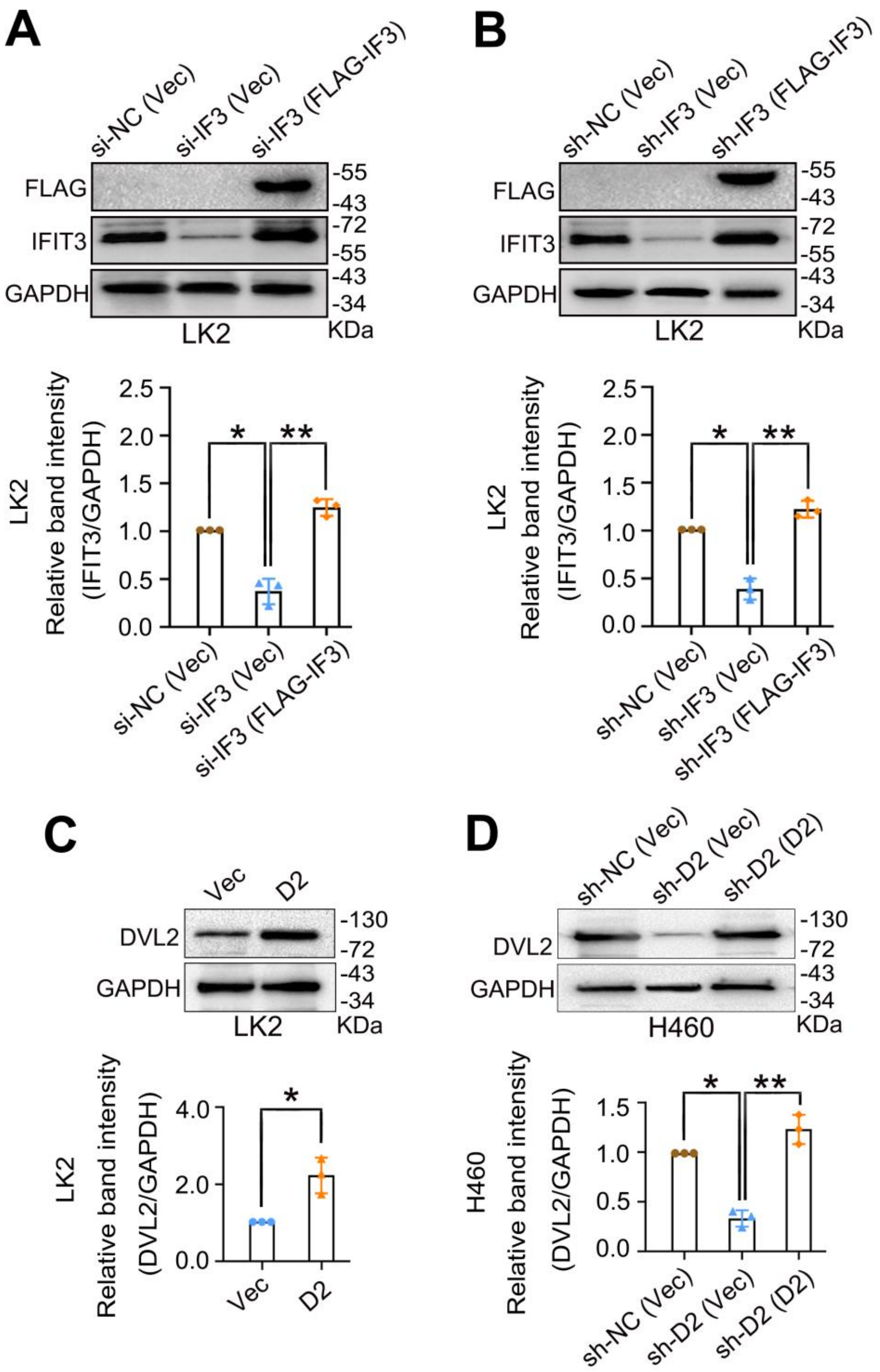

Fig. S2. **Overexpression or knockdown of IFIT3/DVL2. (A–D)** Transfection efficiency. si-NC, siRNA-negative control, a scrambled siRNA was selected as a negative control. si-IF3, siRNA-IFIT3. Vec, vector, the control group of corresponding expression plasmid. FLAG-IF3, transfection of the FLAG-tagged expression plasmid encoding IFIT3. sh-NC, shRNA-negative control, a scrambled shRNA was selected as a negative control. sh-IF3, shRNA-IFIT3. D2, transfection of DVL2 expression plasmid. sh-D2, shRNA-DVL2. si-IF3 (FLAG-IF3), following IFIT3 knockdown with

siRNA, its expression was restored by transfection with the FLAG-tagged expression plasmid encoding IFIT3. sh-IF3 (FLAG-IF3), following IFIT3 knockdown with shRNA, its expression was restored by transfection with the FLAG-tagged expression plasmid encoding IFIT3. sh-D2 (D2), following DVL2 knockdown with shRNA, its expression was restored by transfection with DVL2 expression plasmid. Band intensity of IFIT3/DVL2 was quantified and normalized against that of GAPDH. Data were analyzed by one-way ANOVA with Tukey's post hoc test for (A–B, D) and two-tailed unpaired Student's t-test for (C). Error bars indicated mean ± SEM (n = 3). *p <0.05, **p <0.01.

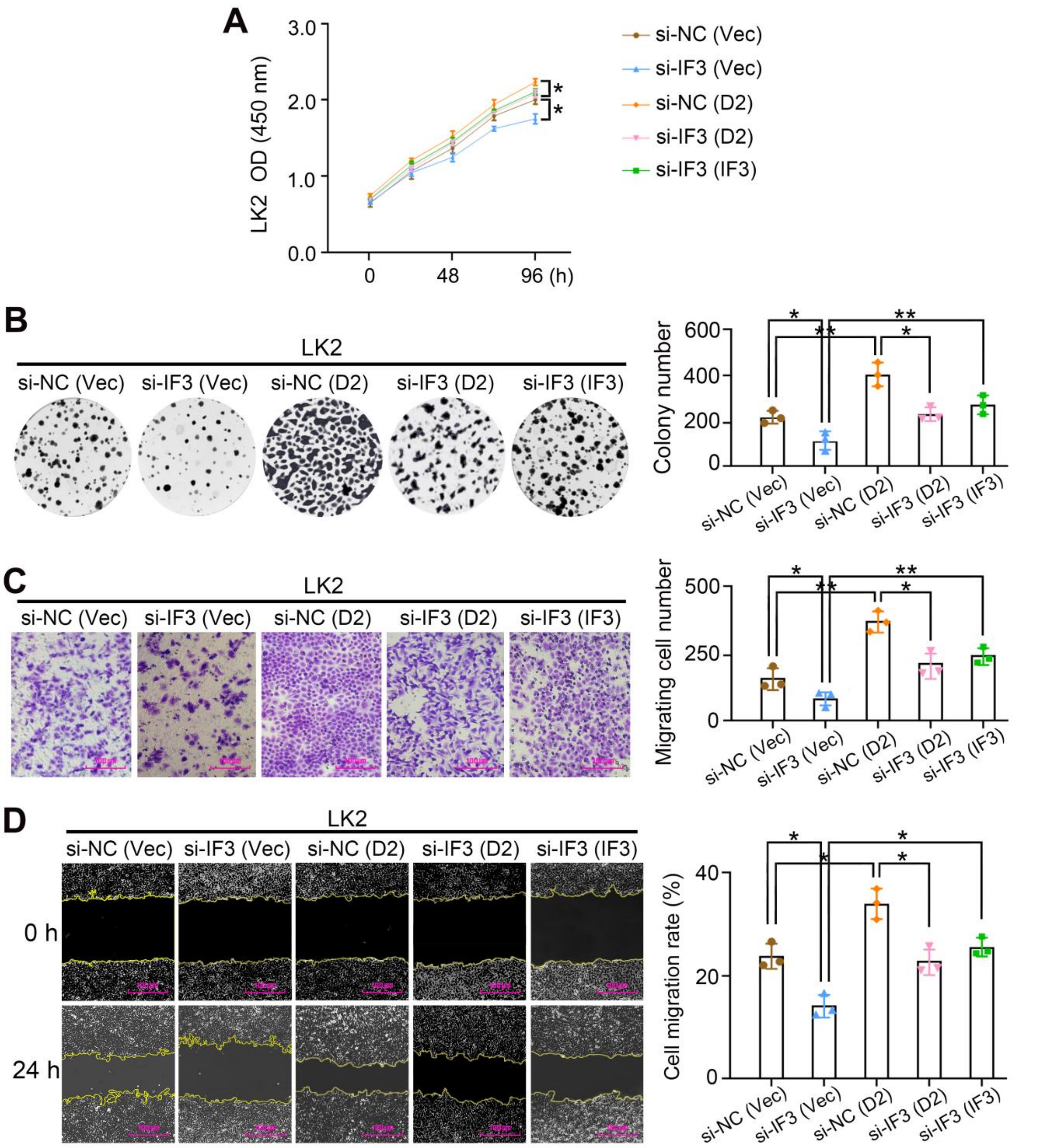

Fig. S3. **Effect of IFIT3 on the biological behavior of LK2 cells. (A)** Viability. **(B)** Colony formation. **(C)** Transwell migration. **(D)** Wound healing. (B–D), the representative images (scale bar, 100 μm) and statistical analysis graphs. LK2 cells were co-transfected with siRNA and plasmid. si-NC, siRNA-negative control, a scrambled siRNA was selected as a negative control. si-IF3, siRNA-IFIT3. Vec, vector, the control group of corresponding expression plasmid. IF3, transfection

of IFIT3 expression plasmid. D2, transfection of DVL2 expression plasmid. Data were analyzed by two-way ANOVA with Šídák's post hoc test for (A) and one-way ANOVA with Tukey's post hoc test for (B–D). Error bars indicated mean ± SD (n = 3) for (A), and mean ± SEM (n = 3) for (B–D). *p <0.05, **p <0.01.

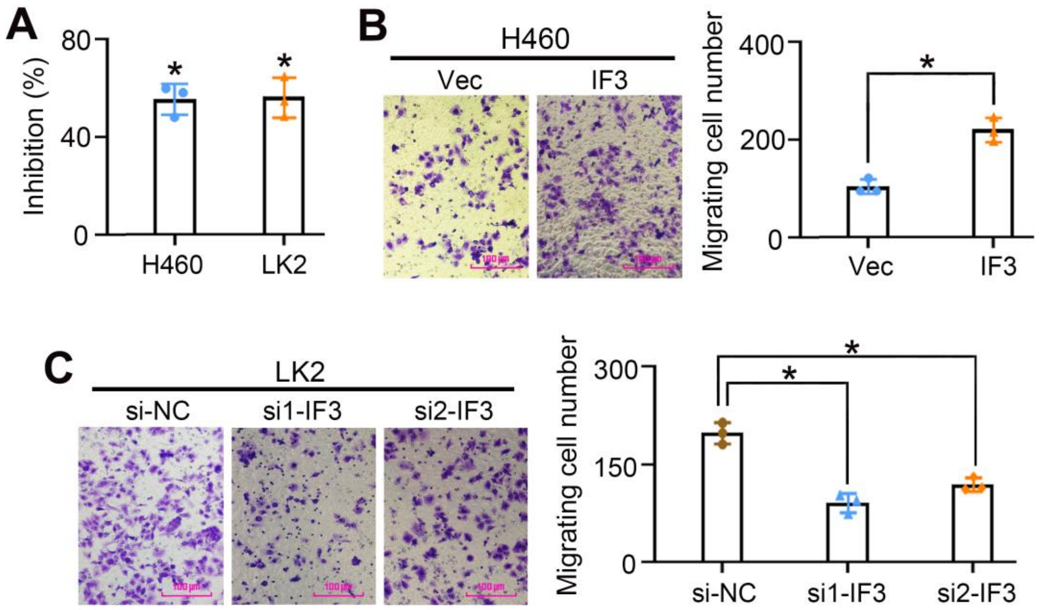

Fig. S4. **Effect of IFIT3 on the biological behavior of LUSC and LCLC cells. (A)** Growth inhibitory effect of mitomycin C (100 μM, 24 h) on H460 and LK2 cells. The graphs are expressed as the percentage growth inhibition with respect to untreated cells. After the cells were treated with mitomycin C (100 μM) for 24 h, IFIT3 siRNA or IFIT3 expression plasmid was transfected to the cells. **(B–C)** Transwell migration of H460 and LK2 cells (scale bar, 100 μm). Vec, vector, the control group of IFIT3 expression plasmid. IF3, transfection of IFIT3 expression plasmid. si1-IF3 and si2-IF3, two pairs of siRNAs for IFIT3. si-NC, siRNA-negative control, a scrambled siRNA was selected as a negative control. Data were analyzed by two-tailed unpaired Student's t-test for (A–B) and one-way ANOVA with Dunnett's post hoc test for (C). Error bars indicated mean ± SEM (n = 3). *$p < 0.05$.

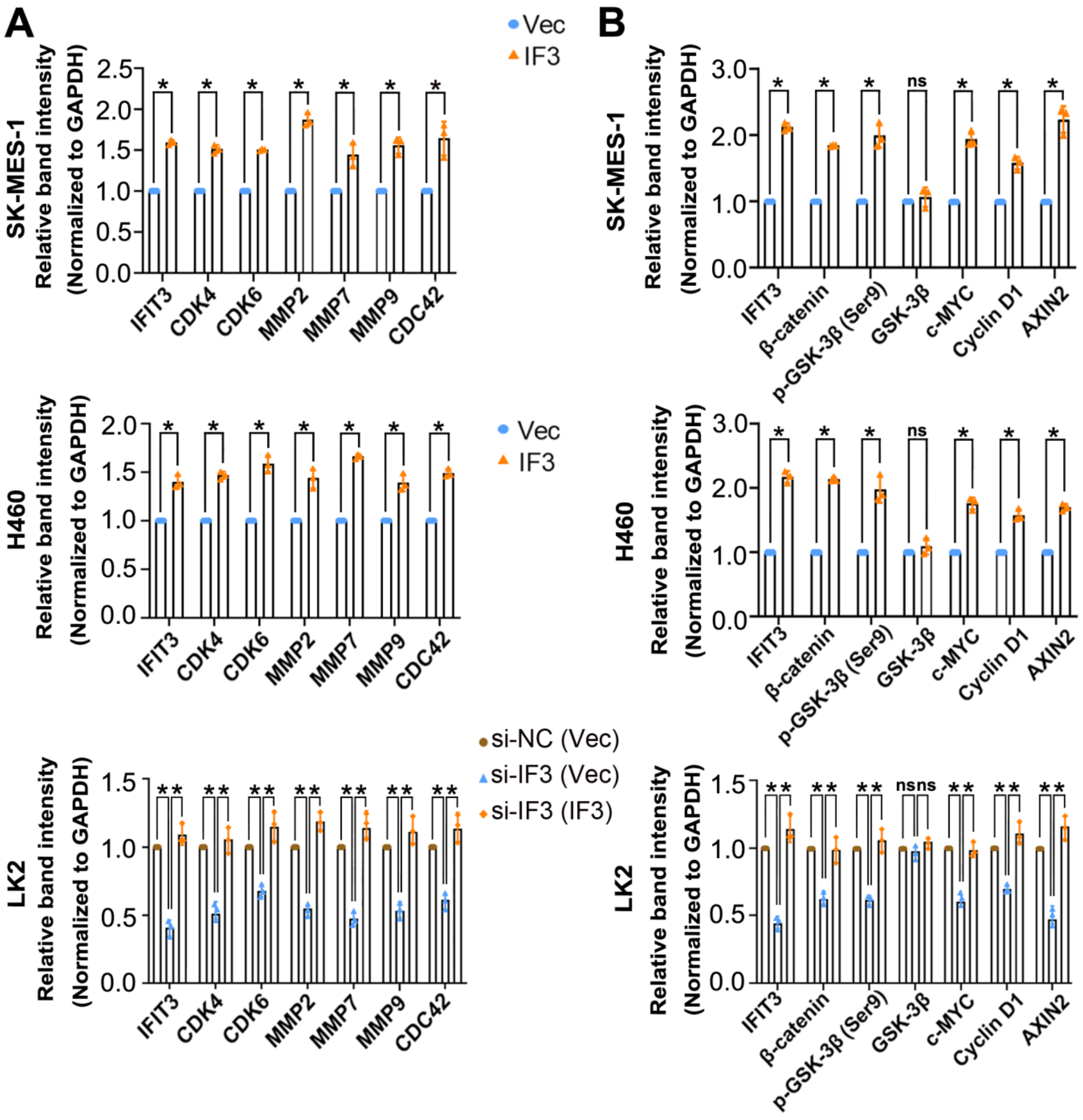

Fig. S5. **The quantification of band intensity (A) in Fig. 3A, and (B) in Fig. 3B.** Band intensity was quantified and normalized against that of GAPDH. Vec, vector, the control group of IFIT3 expression plasmid. IF3, transfection of IFIT3 expression plasmid. si-IF3, siRNA-IFIT3. si-NC, siRNA-negative control, a scrambled siRNA was selected as a negative control. si-IF3 (IF3), following IFIT3 knockdown with siRNA, its expression was restored by transfection with IFIT3 expression plasmid. Data were analyzed by two-tailed unpaired Student's t-test (SK-MES-1 and

H460 cells) and one-way ANOVA with Tukey's post hoc test (LK2 cell). Error bars indicated mean ± SEM (n = 3). *p <0.05. ns, no significance.

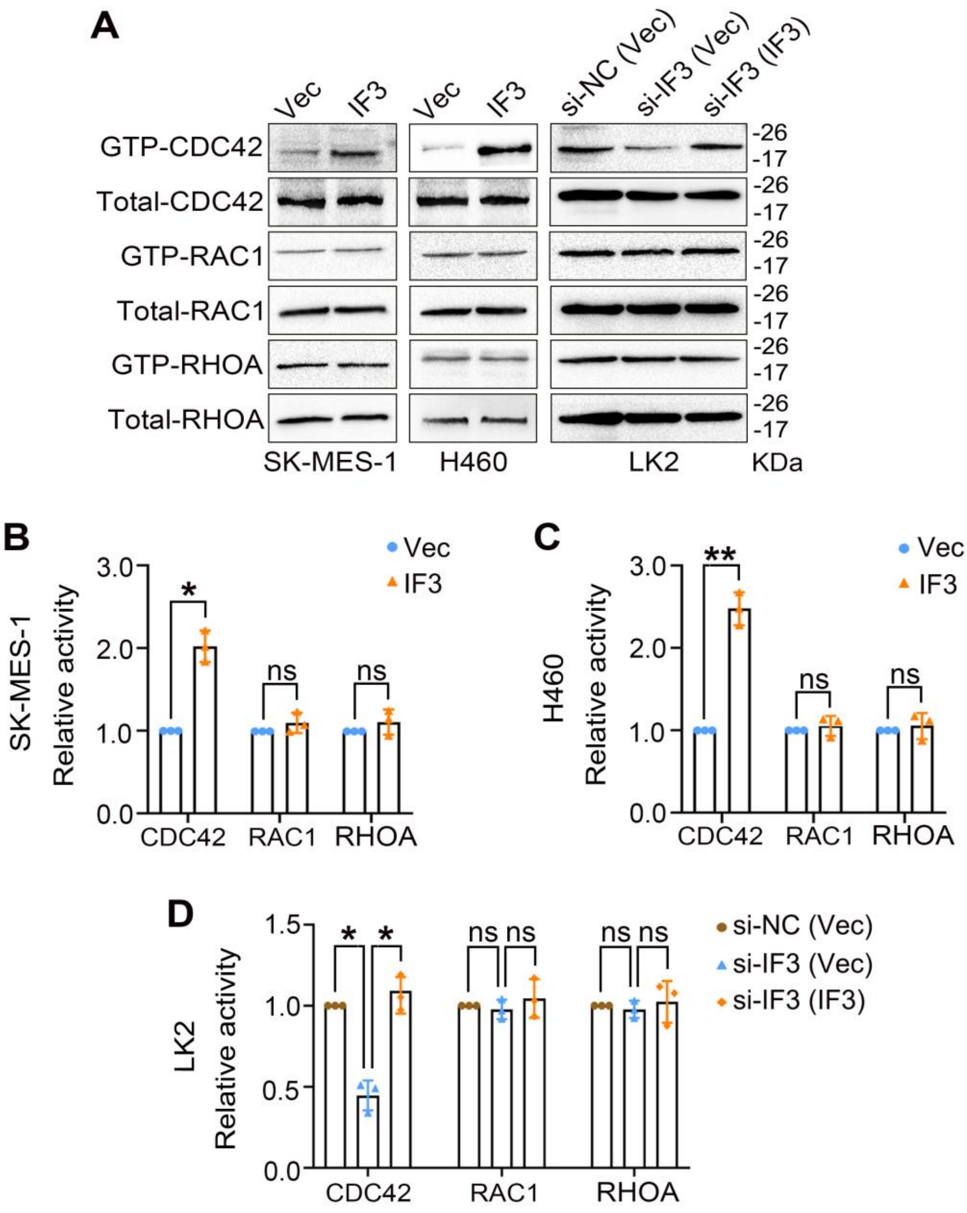

Fig. S6. Effect of IFIT3 on RHO-family GTPase activation. (A) Western blot analysis. **(B–D)** Quantitative analysis of band intensities for (A). Relative CDC42, RAC1, or RHOA activity is the ratio of active CDC42, RAC1, or RHOA divided by total CDC42, RAC1, or RHOA normalized to the untreated control, respectively. Vec, vector, the control group of the IFIT3 expression plasmid. IF3, transfection of IFIT3 expression plasmid. si-NC, siRNA-negative control, a scrambled siRNA was selected as a negative control. si-IF3, siRNA-IFIT3. si-IF3 (IF3), following IFIT3 knockdown with siRNA, its expression was restored by transfection with IFIT3 expression plasmid. Data were analyzed by two-tailed unpaired Student’s t-test for (B–C) and one-way ANOVA with Tukey's post hoc test for (D). Error bars indicated mean ± SEM (n = 3). *p <0.05, **p <0.01. ns, no significance.

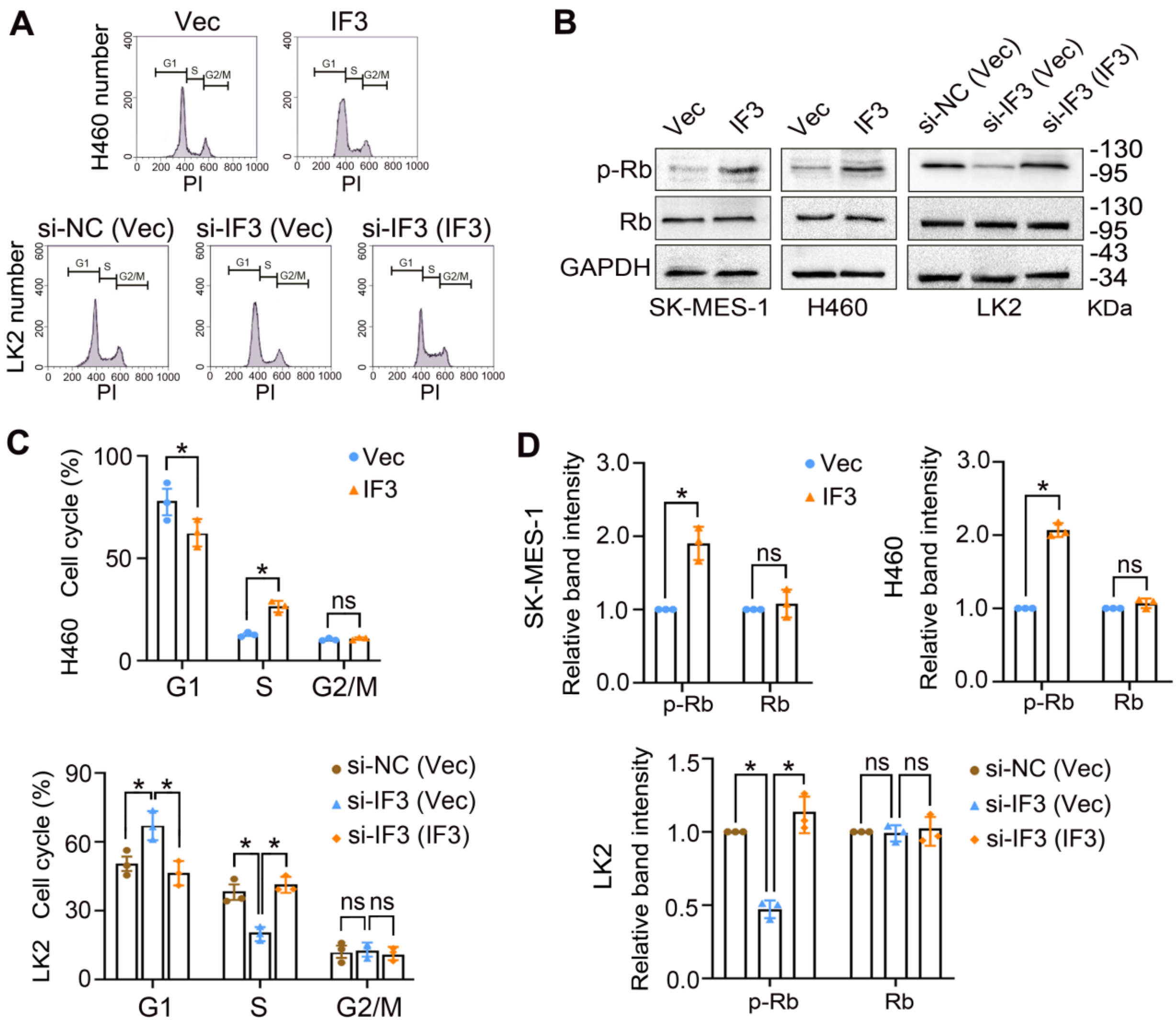

Fig. S7. Effect of IFIT3 on cell cycle. (A, C) Flow cytometry was used to detect the regulation of cell cycle after IFIT3 overexpression or knockdown. **(B, D)** Western blot analysis was used to detect the expression levels of Rb and p-Rb (S780 phosphorylation of Rb). GAPDH was an internal control. Quantitative analysis of band intensities was provided. Vec, vector, the control group of the IFIT3 expression plasmid. IF3, transfection of IFIT3 expression plasmid. si-NC, siRNA-negative control, a scrambled siRNA was selected as a negative control. si-IF3, siRNA-IFIT3. si-IF3 (IF3), following IFIT3 knockdown with siRNA, its expression was restored by transfection with IFIT3 expression plasmid. Data were analyzed by two-tailed unpaired Student's t-test (H460 and SK-MES-1 cells) and one-way ANOVA with Tukey's post hoc test (LK2 cell) for (C–D). Error bars indicated mean ± SEM (n = 3). *p <0.05. ns, no significance.

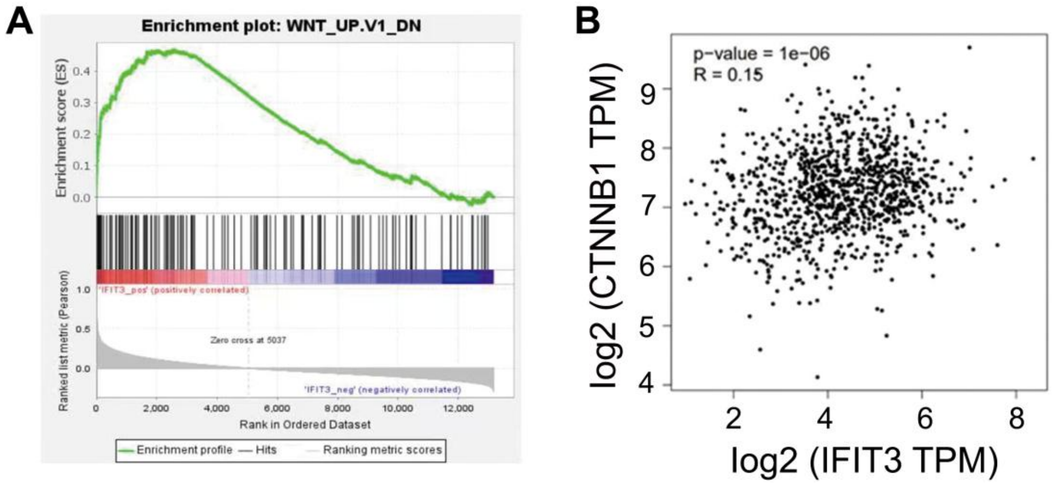

Fig. S8. **Bioinformatics analysis. (A)** Gene set enrichment analysis. **(B)** Spearman correlation analysis using the GEPIA database.

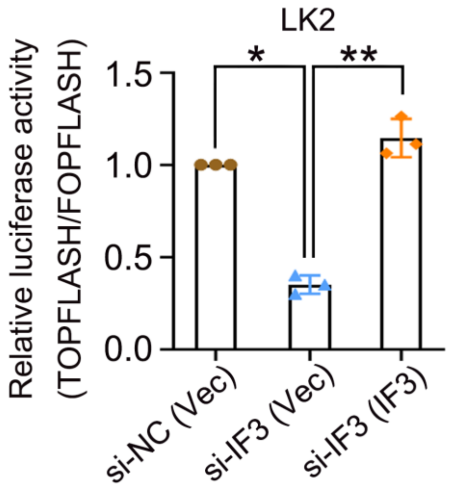

Fig. S9. **Impact of IFIT3 on β-catenin-dependent canonical WNT signaling activity.** Dual-luciferase assay. Vec, vector, the control group of IFIT3 expression plasmid. IF3, transfection of IFIT3 expression plasmid. si-IF3, siRNA-IFIT3. si-NC, siRNA-negative control, a scrambled siRNA was selected as a negative control. si-IF3 (IF3), following IFIT3 knockdown with siRNA, its expression was restored by transfection with IFIT3 expression plasmid. Data were analyzed by one-way ANOVA with Tukey's post hoc test. Error bars indicated mean ± SEM (n = 3). *p <0.05, **p <0.01.

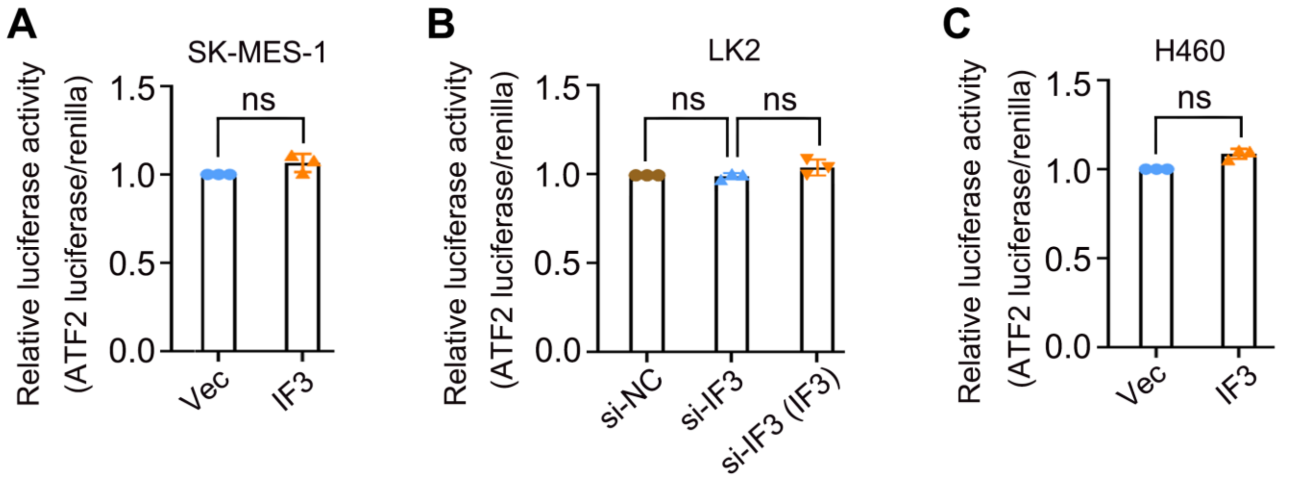

Fig. S10. **Impact of IFIT3 on β-catenin-independent noncanonical WNT signaling activity.** (**A–C**) Dual-luciferase assay. Vec, vector, the control group of IFIT3 expression plasmid. IF3, transfection of IFIT3 expression plasmid. si-IF3, siRNA-IFIT3. si-NC, siRNA-negative control, a scrambled siRNA was selected as a negative control. si-IF3 (IF3), following IFIT3 knockdown with siRNA, its expression was restored by transfection with IFIT3 expression plasmid. Data were analyzed by two-tailed unpaired Student's t-test for (A and C) and one-way ANOVA with Tukey's post hoc test for (B). Error bars indicated mean ± SEM ($n = 3$). ns, no significance.

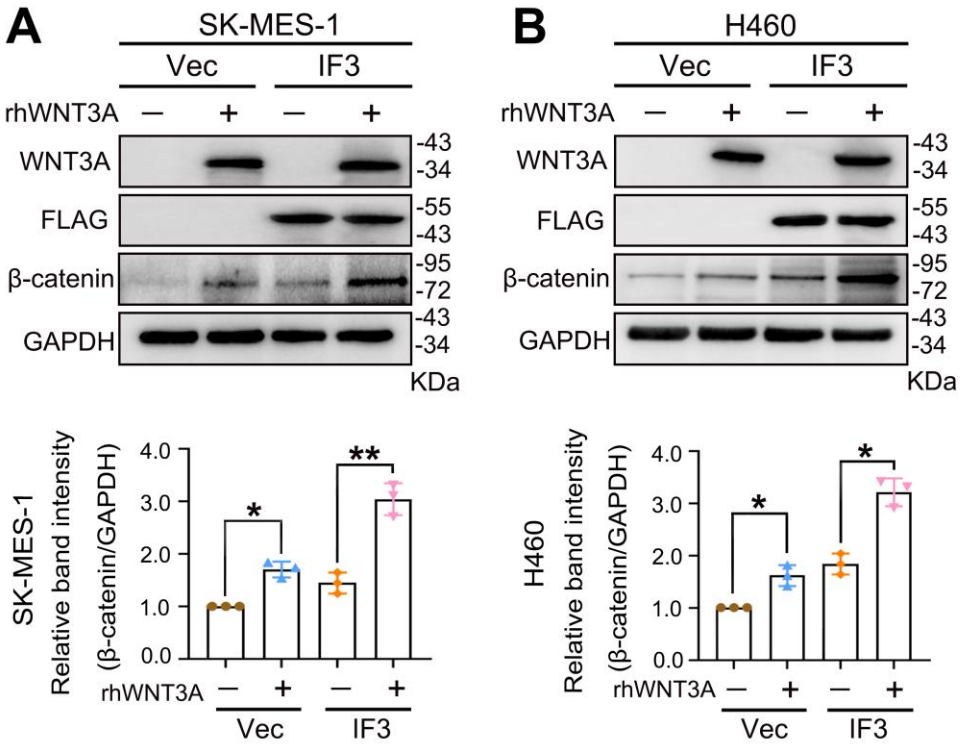

Fig. S11. The read out of IFIT3-transfection elevated canonical WNT signaling. (A–B) Western blot analysis. Band intensity was quantified and normalized against that of GAPDH. rhWNT3A, the cells were treated with recombinant human WNT3A protein (100 ng/mL) for 12 h. Vec, vector, the control group of IFIT3 expression plasmid. IF3, transfection of the expression plasmid encoding FLAG-tagged IFIT3. Data were analyzed by one-way ANOVA with Tukey's post hoc test. Error bars indicated mean ± SEM (n = 3). *p <0.05, **p <0.01.

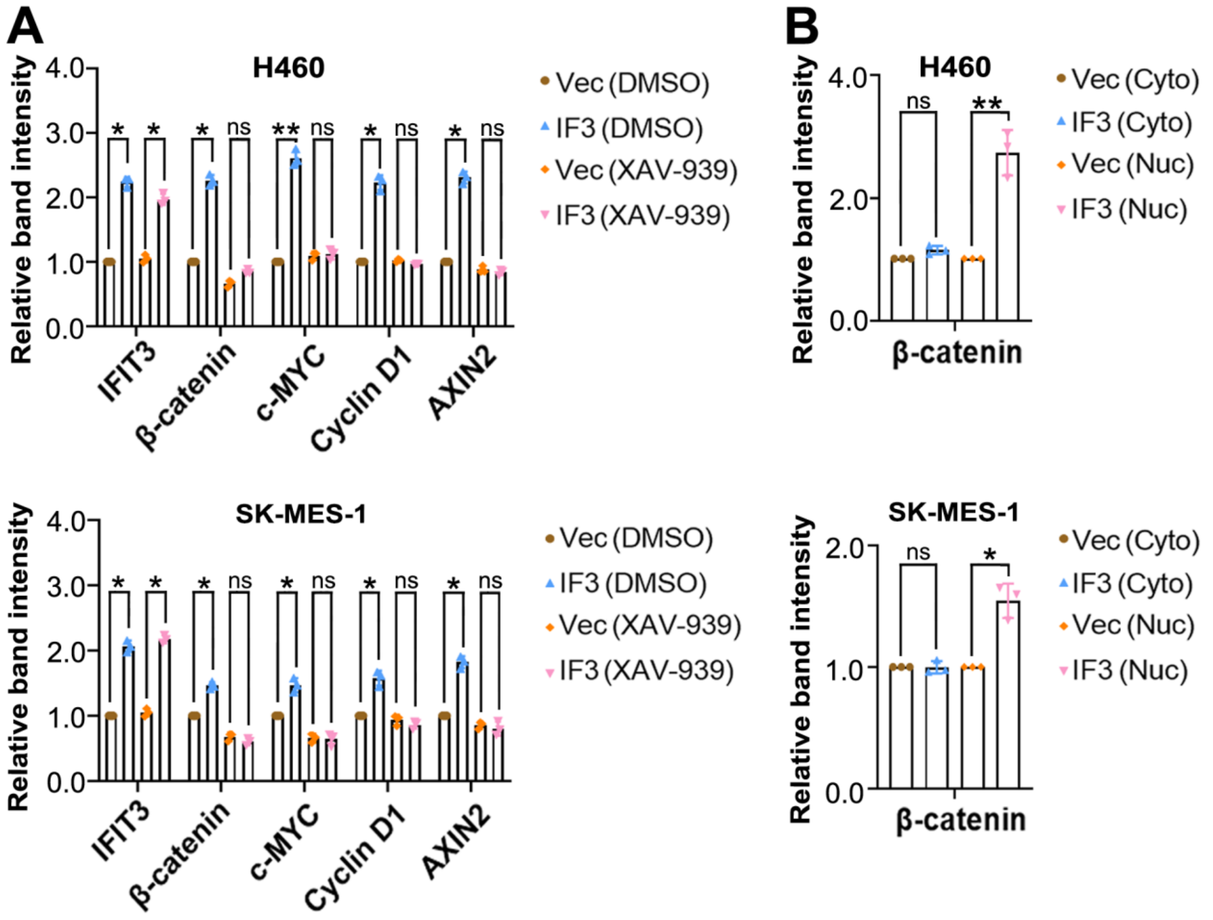

Fig. S12. **The quantification of band intensity (A) in Fig. 4A, and (B) in Fig. 4C.** Band intensity was quantified and normalized against that of GAPDH (A), α-tubulin (B, cyto) or Lamin B1 (B, Nuc). Vec, vector, the control group of the IFIT3 expression plasmid. IF3, transfection of IFIT3 expression plasmid. After transfection with IFIT3 expression plasmid, cells were treated for 24 h with dimethyl sulfoxide (DMSO) (10 μM; IFIT3+DMSO group) or XAV-939 (10 μM; IFIT3+XAV-939 group). Data were analyzed by one-way ANOVA with Tukey's post hoc test. Error bars indicated mean ± SEM (n = 3). *p <0.05, **p <0.01. ns, no significance.

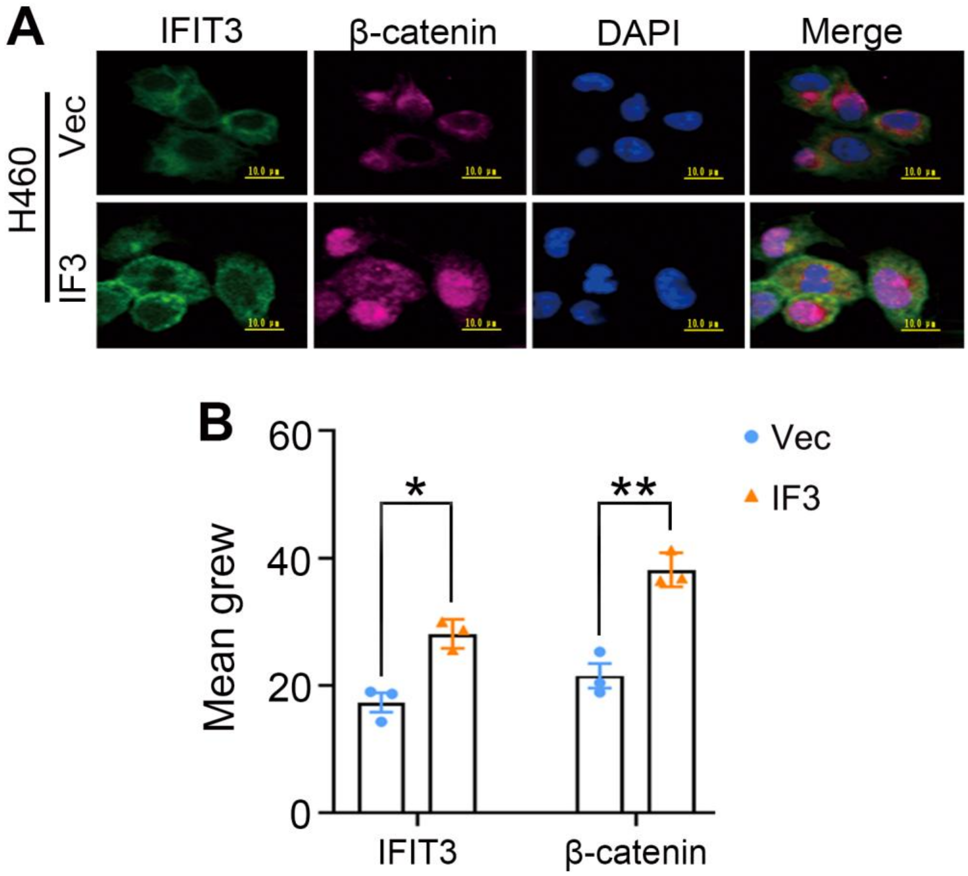

Fig. S13. **Immunofluorescence analysis of β-catenin nuclear translocation. (A)** H460 cells were transfected with IFIT3 expression plasmid. Vec, vector, the control group of IFIT3 expression plasmid. IF3, transfection of IFIT3 expression plasmid. The images shown are representative of three independent experiments. Scale bar, 10 μm. **(B)** Quantification of average fluorescence intensity was provided. Data were analyzed by two-tailed unpaired Student's t-test. Error bars indicated mean ± SEM (n = 3). *p <0.05, **p <0.01.

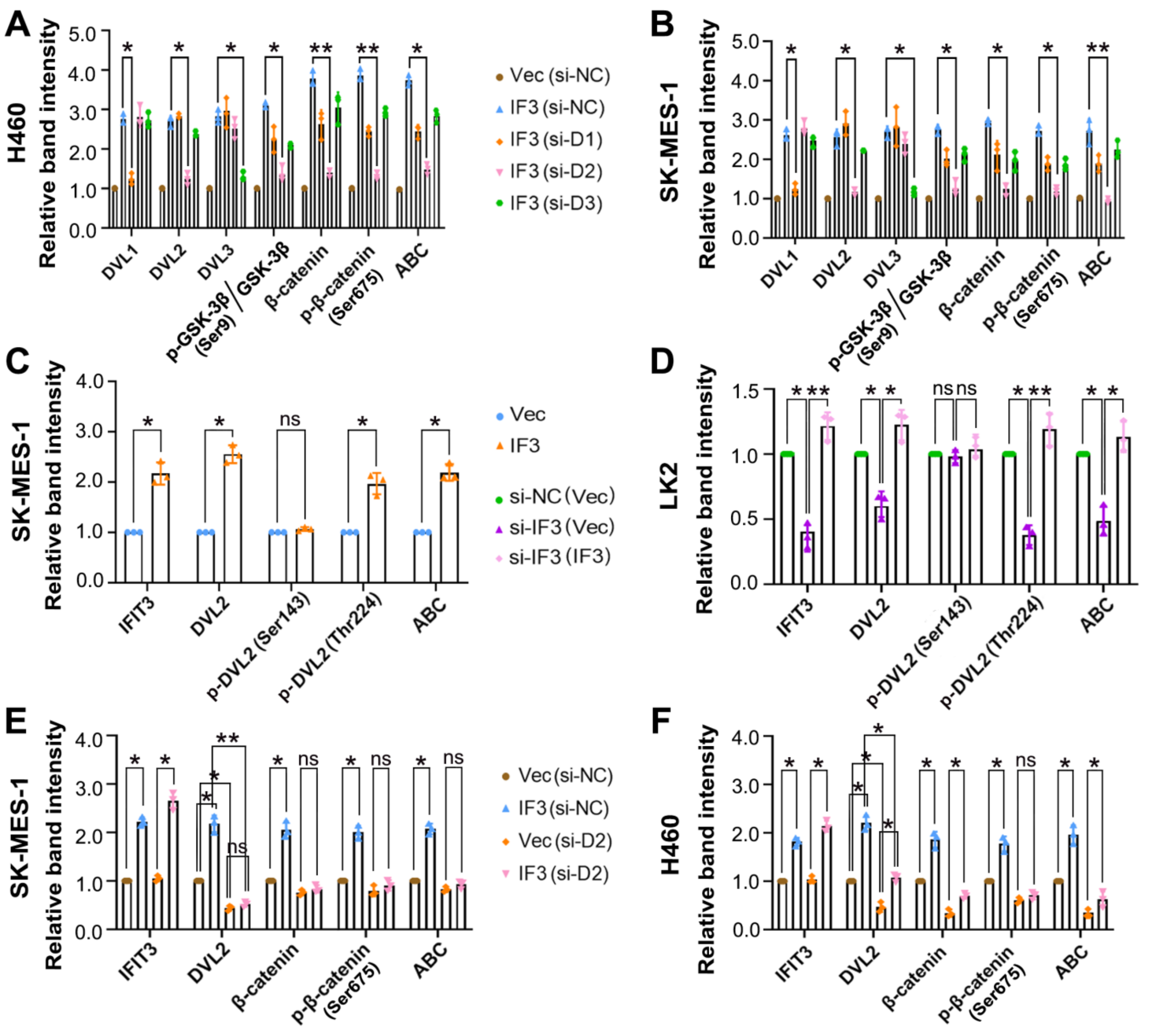

Fig. S14. **The quantification of band intensity (A–B) in Fig. 5C, (C–D) in Fig. 5E, and (E–F) in Fig. 5F.** Band intensity was quantified and normalized against that of GAPDH (p-GSK-3β was quantified and normalized against that of GSK-3β). Vec, vector, the control group of the IFIT3 expression plasmid. IF3, transfection of IFIT3 expression plasmid. si-D1, siRNA-DVL1. si-D2, siRNA-DVL2. si-D3, siRNA-DVL3. si-IF3, siRNA-IFIT3. si-NC, siRNA-negative control, a scrambled siRNA was selected as a negative control. si-IF3 (IF3), following IFIT3 knockdown with siRNA, its expression was restored by transfection with IFIT3 expression plasmid. Data were analyzed by one-way ANOVA with Tukey's post hoc test for (A–B, D–F) and two-tailed unpaired Student's t-test for (C). Error bars indicated mean ± SEM (n = 3). *p <0.05, **p <0.01. ns, no significance.

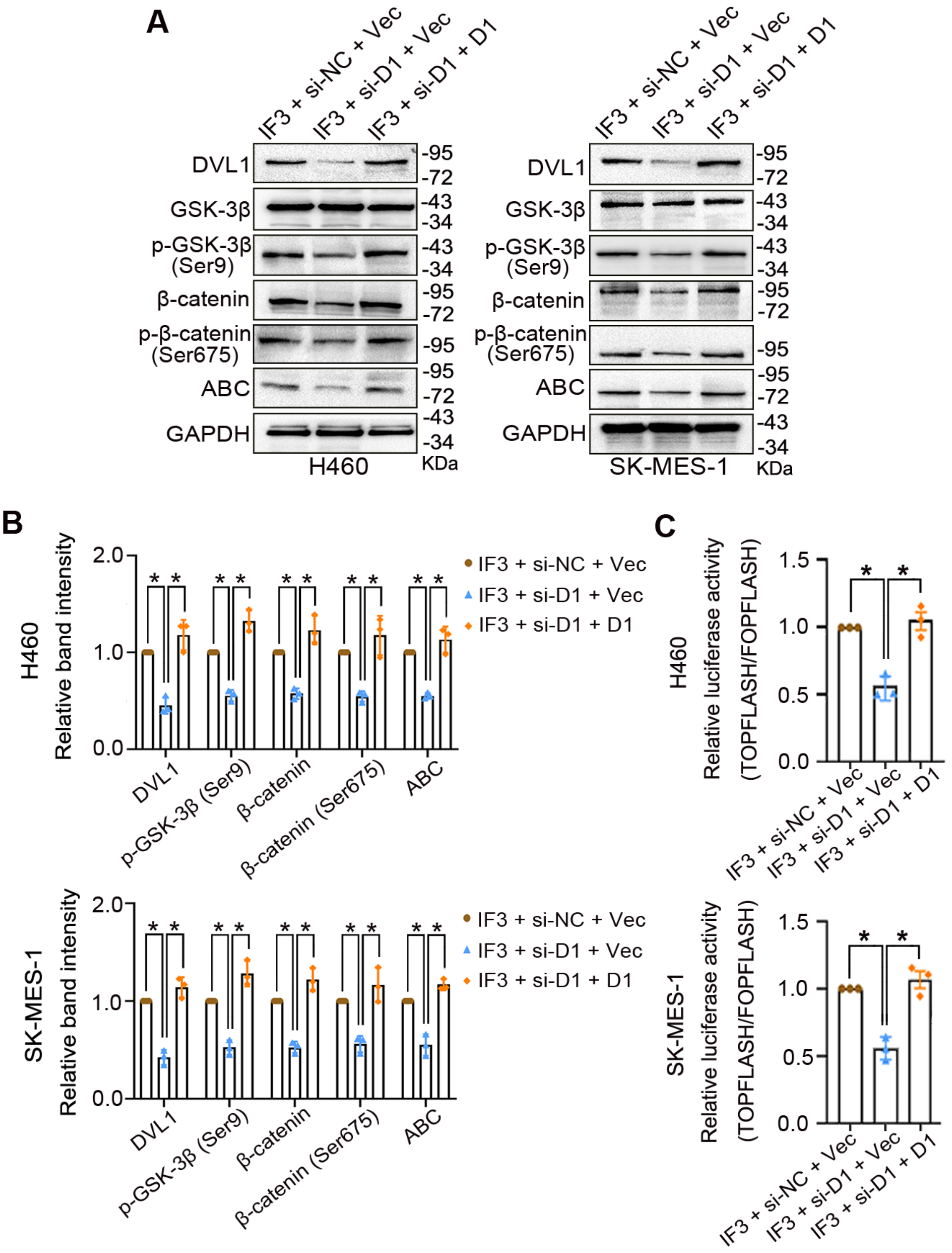

Fig. S15. The rescue experiment for DVL1 knockdown on IFIT3 activating canonical WNT signaling. (A) Western blot analysis. GAPDH was an internal control. **(B)** Quantitative analysis of band intensities. **(C)** Dual-luciferase assay. p-GSK-3β (Ser9), the serine 9 phosphorylation of GSK-3β. p-β-catenin (Ser675), the serine 675 phosphorylation of β-catenin. ABC, active β-catenin. H460 and SK-MES-1 cells were co-transfected with siRNA and plasmid. Vec, vector, the control

group of corresponding expression plasmid. IF3, transfection of IFIT3 expression plasmid. D1, transfection of DVL1 expression plasmid. si-D1, siRNA-DVL1. si-NC, siRNA-negative control, a scrambled siRNA was selected as a negative control. Data were analyzed by one-way ANOVA with Tukey's post hoc test. Error bars indicated mean ± SEM (n = 3). *$p < 0.05$.

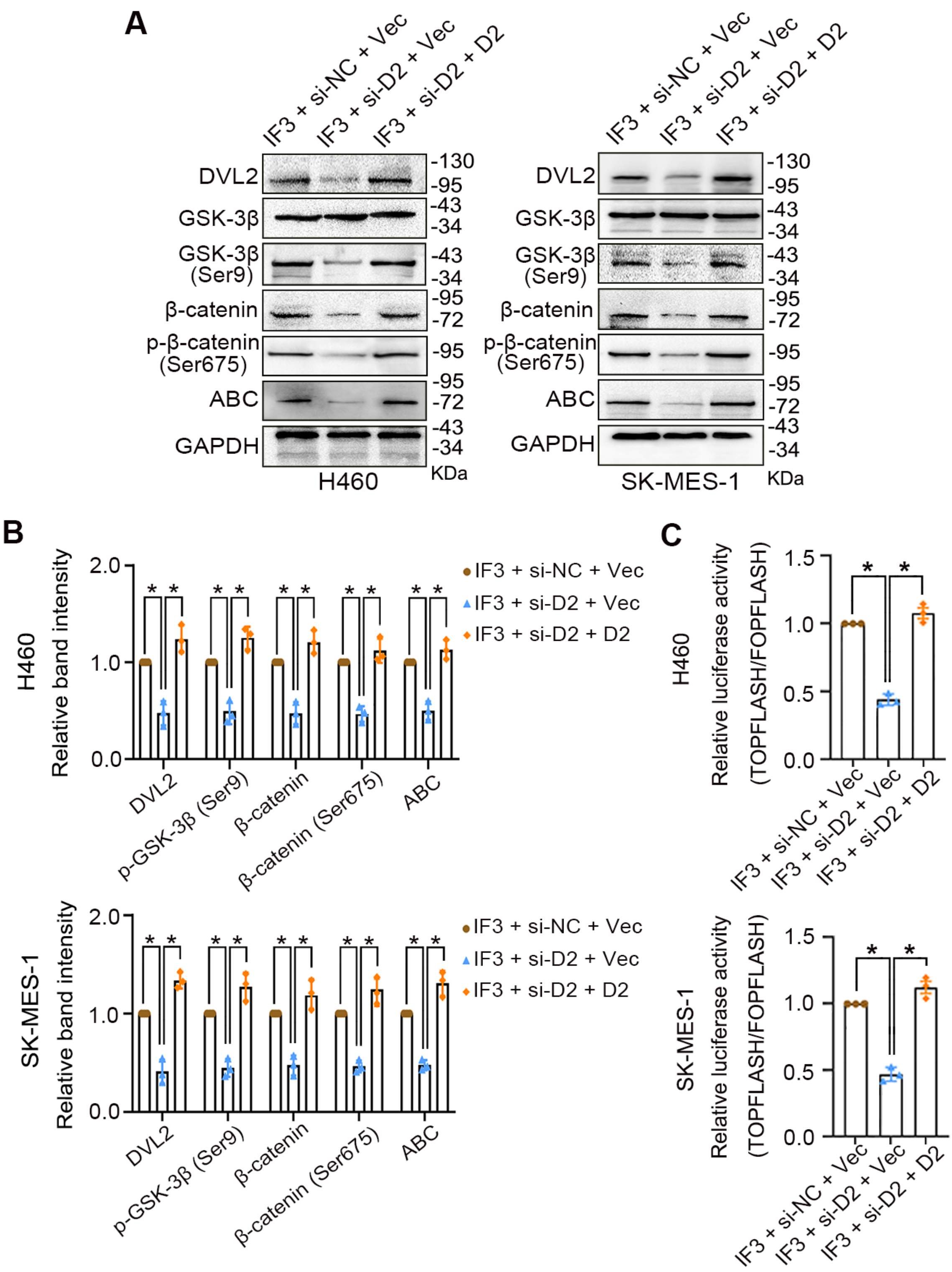

Fig. S16. The rescue experiment for DVL2 knockdown on IFIT3 activating canonical WNT signaling. (A) Western blot analysis. GAPDH was an internal control. **(B)** Quantitative analysis of band intensities. **(C)** Dual-luciferase assay. p-GSK-3β (Ser9), the serine 9 phosphorylation of GSK-3β. p-β-catenin (Ser675), the serine 675 phosphorylation of β-catenin. ABC, active β-catenin. H460 and SK-MES-1 cells were co-transfected with siRNA and plasmid. Vec, vector, the control

group of corresponding expression plasmid. IF3, transfection of IFIT3 expression plasmid. D2, transfection of DVL2 expression plasmid. si-D2, siRNA-DVL2. si-NC, siRNA-negative control, a scrambled siRNA was selected as a negative control. Data were analyzed by one-way ANOVA with Tukey's post hoc test. Error bars indicated mean ± SEM (n = 3). *$p < 0.05$.

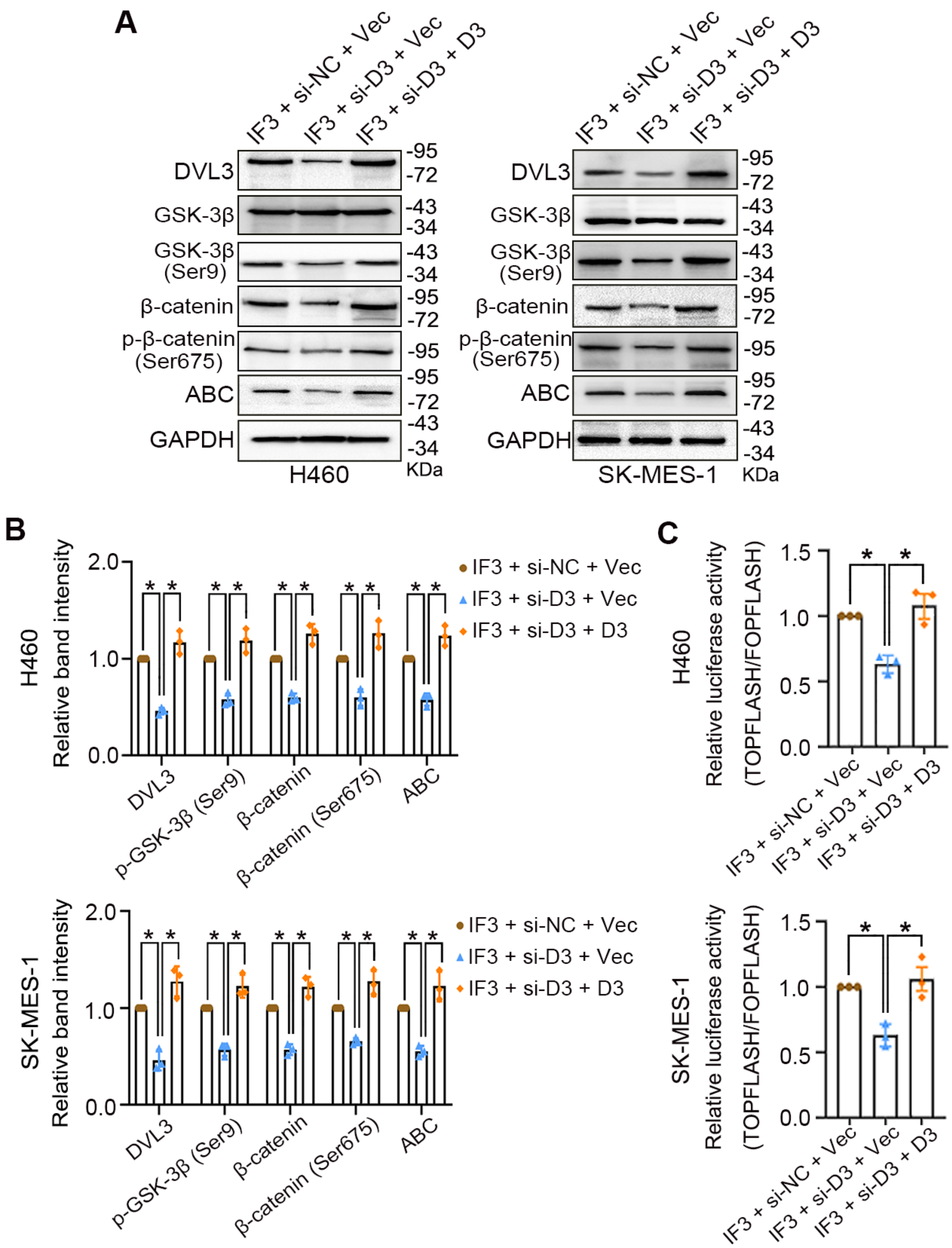

Fig. S17. The rescue experiment for DVL3 knockdown on IFIT3 activating canonical WNT signaling. (A) Western blot analysis. GAPDH was an internal control. **(B)** Quantitative analysis of band intensities. **(C)** Dual-luciferase assay. p-GSK-3β (Ser9), the serine 9 phosphorylation of GSK-3β. p-β-catenin (Ser675), the serine 675 phosphorylation of β-catenin. ABC, active β-catenin. H460 and SK-MES-1 cells were co-transfected with siRNA and plasmid. Vec, vector, the control

group of corresponding expression plasmid. IF3, transfection of IFIT3 expression plasmid. D3, transfection of DVL3 expression plasmid. si-D3, siRNA-DVL3. si-NC, siRNA-negative control, a scrambled siRNA was selected as a negative control. Data were analyzed by one-way ANOVA with Tukey's post hoc test. Error bars indicated mean ± SEM (n = 3). *p <0.05.

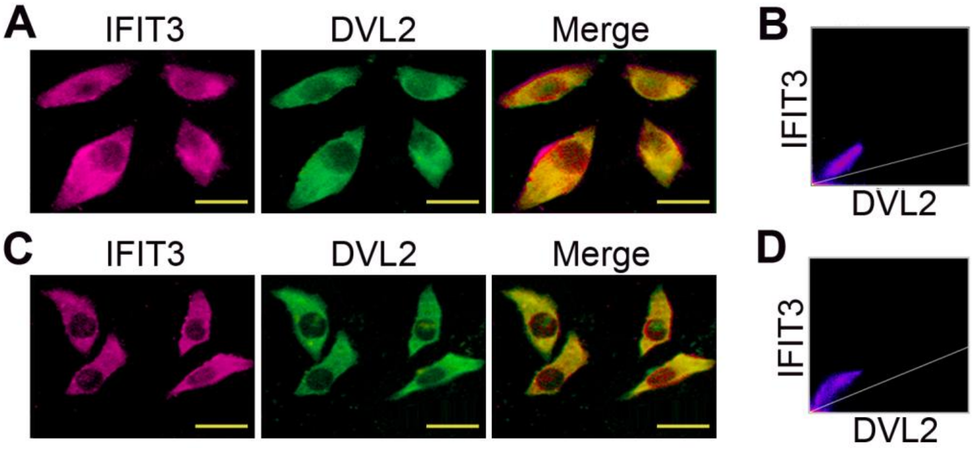

Fig. S18. Colocalization analysis of immunofluorescence images. **(A)** Representative images of colocalization of IFIT3 (red) and DVL2 (green) in SK-MES-1 cell. **(B)** Scatter plot analysis with ImageJ for IFIT3 and DVL2 in SK-MES-1 cell as shown in (A). **(C)** Representative images of colocalization of IFIT3 (red) and DVL2 (green) in H460 cell. **(D)** Scatter plot analysis with ImageJ for IFIT3 and DVL2 in H460 cell as shown in (C). The co-localization analysis shown in (A) and (C) was performed on the original image presented in Fig. 5B. Scale bar, 10 µm.

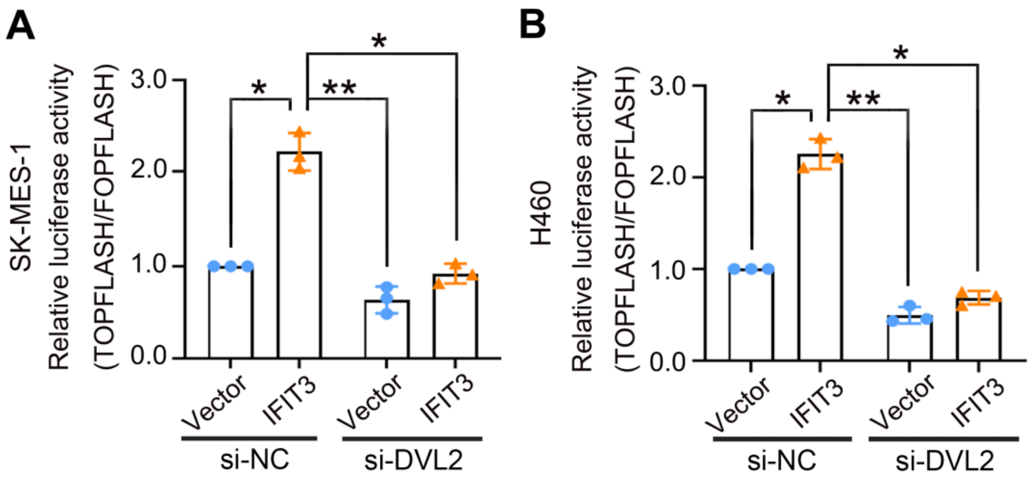

Fig. S19. **Impact of IFIT3 on β-catenin-dependent WNT signaling activity. (A–B)** Dual-luciferase assay. SK-MES-1 and H460 cells were co-transfected of IFIT3 expression plasmid with DVL2 siRNA. Vector is the control group of IFIT3 expression plasmid. IFIT3, transfection of IFIT3 expression plasmid. si-DVL2, siRNA-DVL2. si-NC, siRNA-negative control, a scrambled siRNA was selected as a negative control. Data were analyzed by one-way ANOVA with Tukey's post hoc test. Error bars indicated mean ± SEM (n = 3). *p <0.05, **p <0.01.

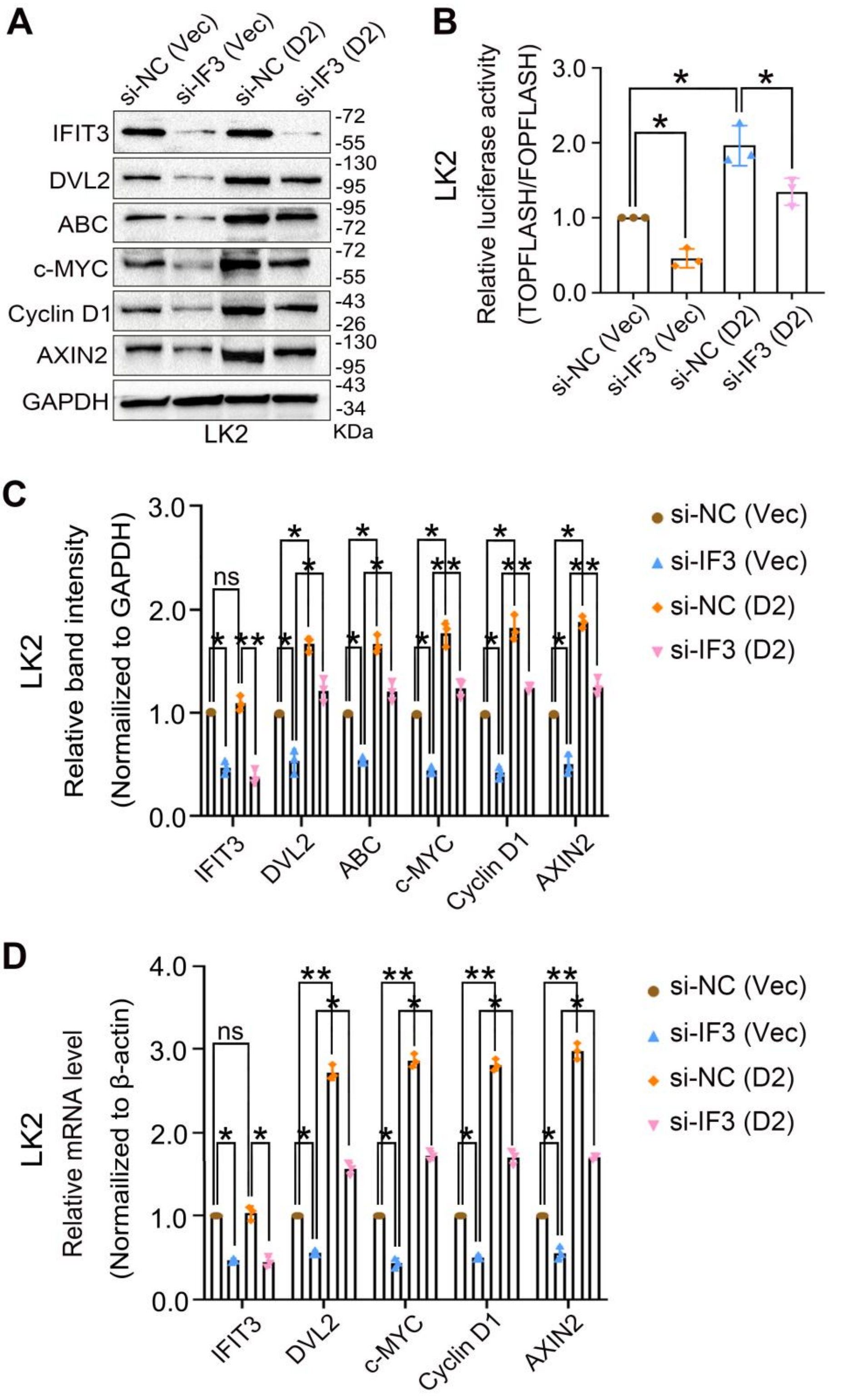

Fig. S20. Effect of DVL2 on the promotion of IFIT3 on canonical WNT signaling. (A) Western blot analysis. GAPDH was an internal control. **(B)** Dual-luciferase assay. **(C)** Quantitative analysis of band intensities for (A). **(D)** Quantitative real-time PCR analysis. β-actin was an internal control. LK2 cells were co-transfected with siRNA and plasmid. Vec, vector, the control group of DVL2 expression plasmid. D2, transfection of DVL2 expression plasmid. si-IF3, siRNA-IFIT3. si-NC,

siRNA-negative control, a scrambled siRNA was selected as a negative control. Data were analyzed by one-way ANOVA with Tukey's post hoc test. Error bars indicated mean ± SEM ($n = 3$). *$p < 0.05$, **$p < 0.01$.

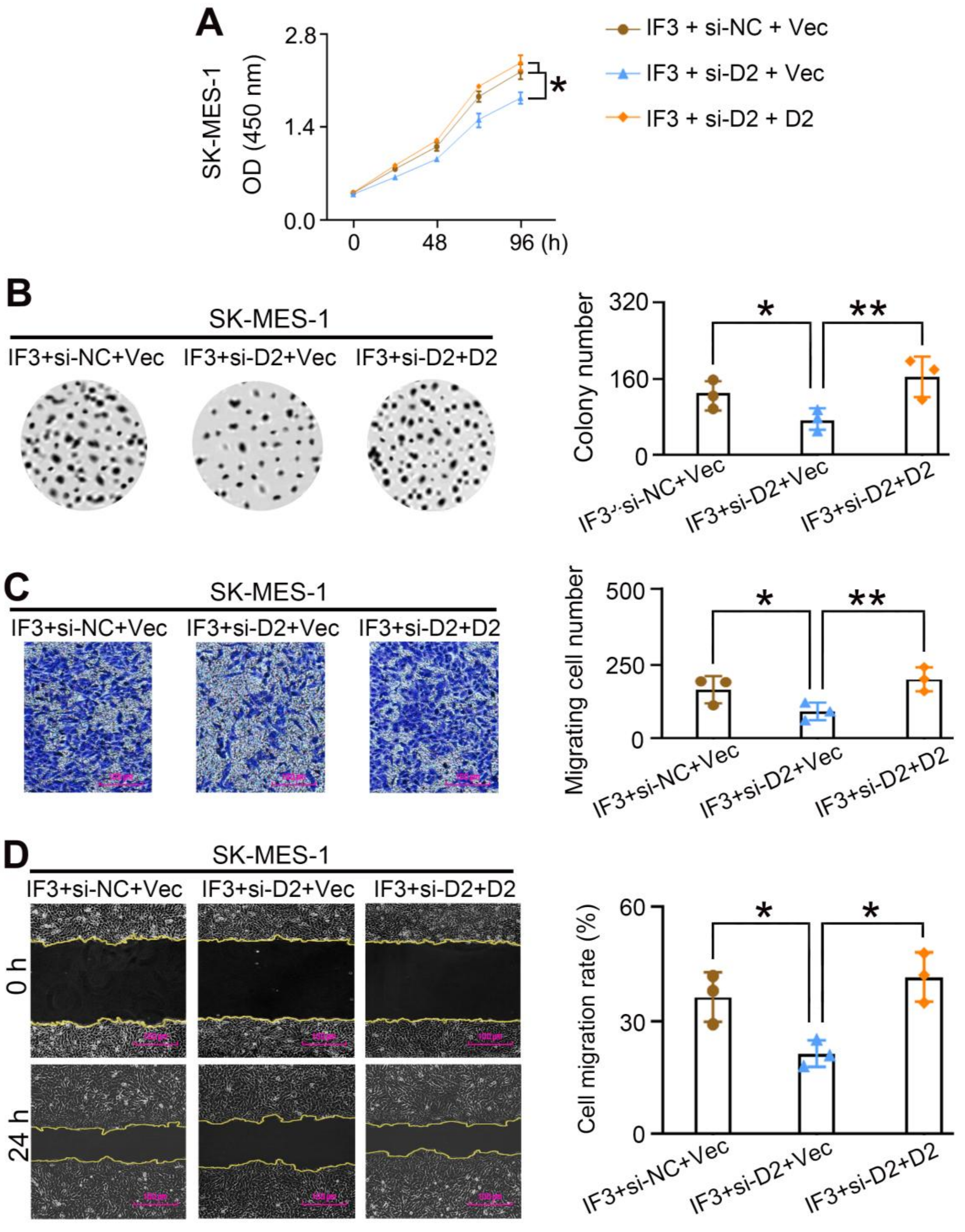

Fig. S21. The rescue experiment for DVL2 knockdown on cell malignancy in SK-MES-1 cell. **(A)** Viability. **(B)** Colony formation. **(C)** Transwell migration. **(D)** Wound healing. (B–D), the representative images (scale bar, 100 μm) and statistical analysis graphs. Vec, vector, the control group of the corresponding expression plasmid. IF3, transfection of IFIT3 expression plasmid. D2, transfection of DVL2 expression plasmid. si-D2, siRNA-DVL2. si-NC, siRNA-negative control, a

scrambled siRNA was selected as a negative control. Data were analyzed by two-way ANOVA with Šídák's post hoc test for (A) and one-way ANOVA with Tukey's post hoc test for (B–D). Error bars indicated mean ± SD (n = 3) for (A), and mean ± SEM (n = 3) for (B–D). *$p < 0.05$, **$p < 0.01$.

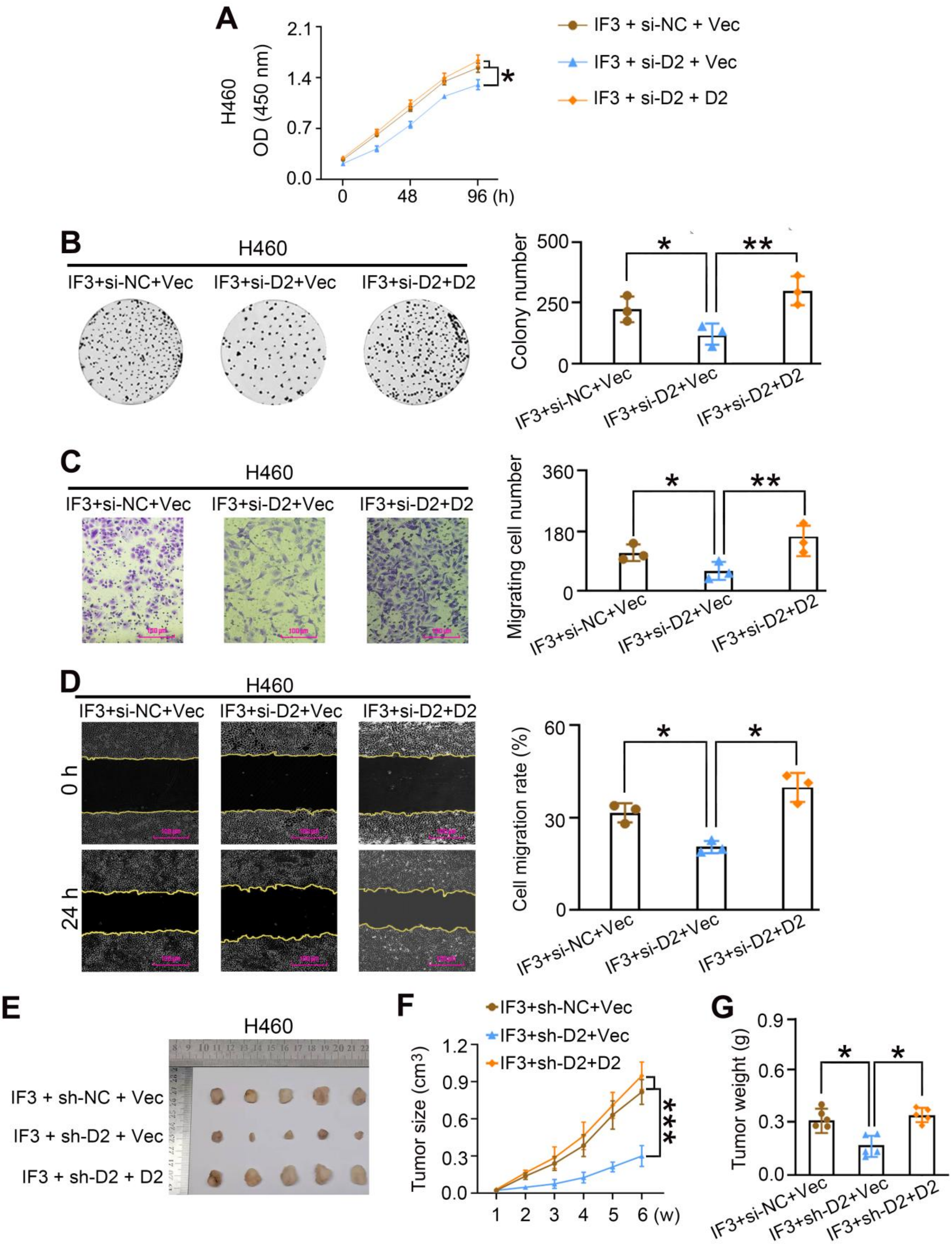

Fig. S22. The rescue experiment for DVL2 knockdown on cell malignancy in H460 cell. (A)

Viability. **(B)** Colony formation. **(C)** Transwell migration. **(D)** Wound healing. **(E–G)** Subcutaneously injected tumors (G418 screening, n = 5). (B–D), the representative images (scale bar, 100 μm) and statistical analysis graphs. Vec, vector, the control group of the corresponding expression plasmid. IF3, transfection of IFIT3 expression plasmid. D2, transfection of DVL2 expression plasmid. si-NC, siRNA-negative control, a scrambled siRNA was selected as a negative control. si-D2, siRNA-DVL2. sh-D2, shRNA-DVL2. sh-NC, shRNA-negative control, a scrambled shRNA was selected as a negative control. Error bars indicated mean ± SD (n = 3) for (A), mean ± SEM (n = 3) for (B–D), and mean ± SD (n = 5 for each group) for (F–G). Data were analyzed by two-way ANOVA with Šídák's post hoc test for (A, F), and one-way ANOVA with Tukey's post hoc test for (B–D, and G). *$p < 0.05$, **$p < 0.01$, ***$p < 0.001$.

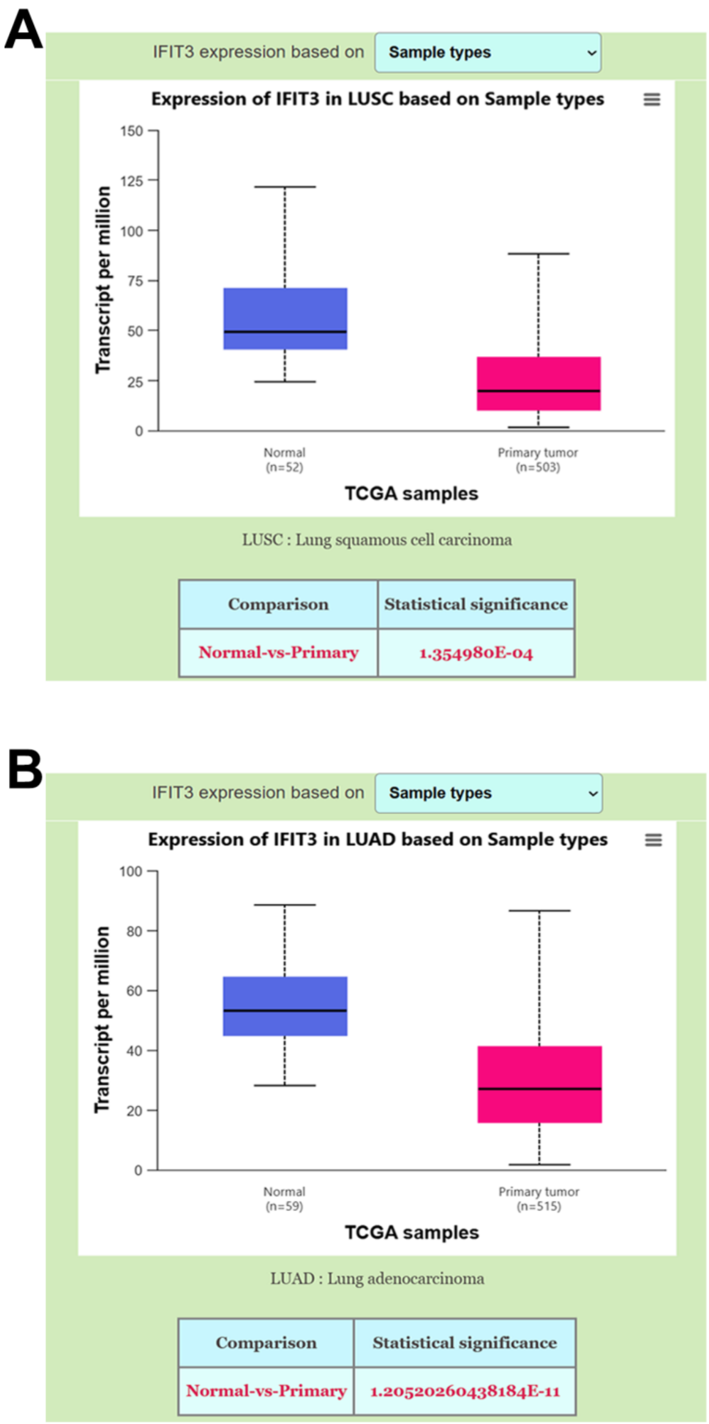

Fig. S23. **Bioinformatics analysis.** The analysis of IFIT3 expression from the TCGA database using UALCAN (University of Alabama at Birmingham) in LUSC samples **(A)** and LUAD samples **(B)**.

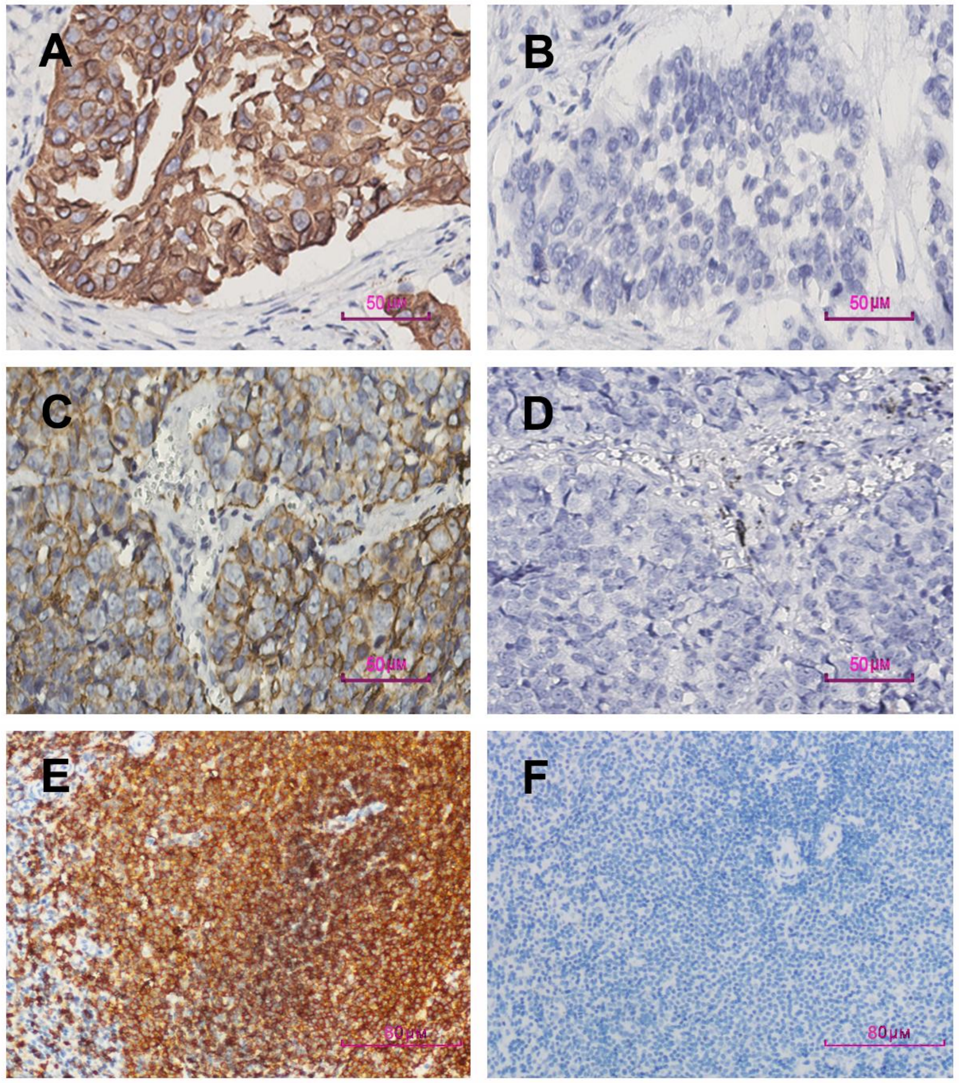

Fig. S24. The validation of antibody for immunohistochemistry. (A–B) LUSC; **(C–D)** LCLC. **(E–F)** Human spleen tissue. A, C, and E: IFIT3 primary antibody. B, D, and F: rabbit IgG was used instead of the primary antibody (A–D, scale bar, 50 μm; E–F, scale bar, 80 μm).

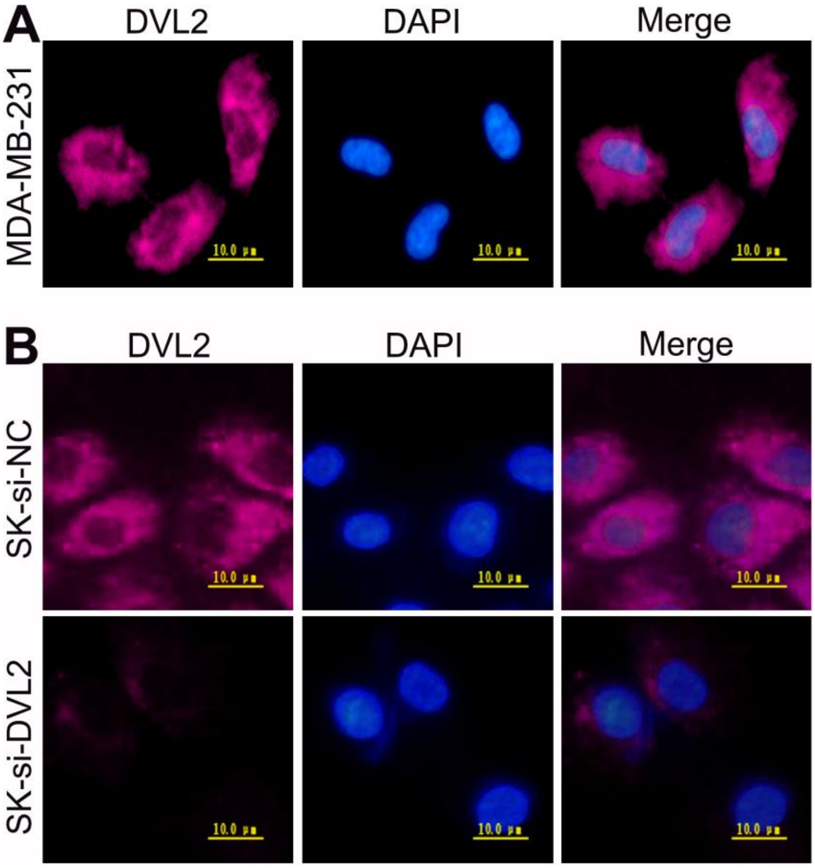

Fig. S25. The validation of DVL2 antibody for immunofluorescence analysis. (A) MDA-MB-231 cells, positive control setting for the validation of DVL2 antibody; **(B)** SK-MES-1 cells, transfection of siRNA-DVL2 (SK-si-DVL2) and siRNA-negative control (SK-si-NC). (A–B, scale bar, 10 μm).

Supplementary Methods

Establishment of stable cell lines

Lentivirus vectors containing one scramble and three shRNAs for IFIT3/DVL2 were purchased from Genechem (Shanghai, China). The three shRNAs were tested and the shRNA with best inhibition effect of IFIT3 knockdown was selected for subsequent experiment. Lentivirus vectors carrying the IFIT3/DVL2 gene and negative control were also purchased from Genechem (Shanghai, China). The following sequence of shRNA for IFIT3 was the best: GTACCGCCCTGAGATGCTTAGCTAAATCTAAGAGATTTACGTAAGCATTCAGGGCTTTTTG (Sense) and AATCTAAGAACCCTGGAATGCTTAGCTAATACTCTGTAATTTACGTAACGTATCCAGGCGCG (Antisense). The following sequence of shRNA for DVL2 was the best: GGAAGAAATTTCAGATGACTTTGATGAGGTGGATAACATC (Sense) and GCTGGTGTCCTCAGATAATCCAGCTATAGATGTCGATA (Antisense).

NSCLC cells were transfected with lentivirus carrying the IFIT3/DVL2 gene/negative control or lentivirus carrying shRNAs targeting IFIT3/DVL2/scramble to obtain cell lines with the stable overexpression or knockdown of IFIT3/DVL2. Transfection was performed according to the manufacturer’s instructions. Cells were selected by puromycin to produce stable transfected cells. The infection efficiency was confirmed by Western blot analysis.

Reagents

DVL1 expression vector pCS2-MYC-DVL1 and control vector were kindly provided by Dr. Zhenge Luo (Institute of Neuroscience, Shanghai Institutes of Biological Sciences, Chinese Academy of Sciences). DVL2 expression plasmid (RC202233) was purchased from OriGene (Rockville, USA). DVL3 expression construct pMYC-CYTO-DVL3 and control vector were generous gifts from Dr. Jun Liu (Department of Chemistry, The Scripps Research Institute, La Jolla, CA).

Bioinformatics analysis

Gene set enrichment analysis (GSEA) is a computational method to identify all enriched biological pathways found to be associated with IFIT3 gene expression. It was performed in the high-expression

and the low-expression groups compared with the median level of IFIT3 expression respectively. For each analysis, gene set permutation was performed 1000 times. Gene sets with a normal p-value less than 5% and false discovery rate less than 25% were considered statistically significant.

Gene expression profiling interactive analysis (GEPIA) database (http://gepia.cancer-pku.cn/) combines the data of TCGA cancer with the data of CRTx normal tissue. It analyses the association and correlation between two genes. We used this database to analyse the correlation between IFIT3 and WNT genes.

The mRNA expression level of IFIT3 gene in cancer tissues compared to normal tissues was analyzed using the UALCAN database (University of Alabama at Birmingham, https://ualcan.path.uab.edu/index.html).

Dual-luciferase assay

We plated cells in 24-well plate. After incubation for 24 h, we transfected the mixture of 100 ng ATF2 (noncanonical WNT/JNK signaling, Promega, Madison, WI) or 100 ng TOPFLASH and FOPFLASH (β-catenin/TCF mediated transcriptional activity, Addgene, Cambridge, MA, USA) and 10 ng Renilla (Promega) according to the instructions of Lipofectamine 2000 (Invitrogen, Carlsbad, USA). For analysis of WNT3A response, cells were treated with recombinant human WNT3A protein (100 ng/mL, R&D systems, Cat# 5036-WN-500/CF) for 12 h prior to dual-luciferase assay. This was used as positive control for β-catenin/TCF mediated transcriptional activity. Cells were treated with the indicated factors in addition to the transfection of corresponding luciferase reporter gene vector. After incubation for 48 h, dual-luciferase assay system (Promega) was performed to measure the luciferase activity.

TOPFLASH/FOPFLASH was used to measure β-catenin/TCF mediated transcriptional activity. ATF2 luciferase was used to measure the activity of the noncanonical WNT/JNK signaling and the values of various luciferase activities were normalized with Renilla activity. We repeated the experiments in triplicate.

Flow cytometry

Cell cycle distributions were determined by flow cytometry. H460 and LK2 cells were treated with

IFIT3 overexpression or knockdown. At incubation time, cells were harvested and fixed in 70% ethanol at 4 °C overnight. Cells were washed thrice with PBS and stained with propidium iodide (PI) at room temperature (22–25 °C) for 30 min in the dark. The cell cycle phases were quantified by FAC Sort flow cytometry (BD Biosciences, California, USA).

RAC1/CDC42/RHOA GTPase activation assays

The RAC1/CDC42 Activation Assay Kit (Sigma-Aldrich, St. Louis, MO, USA) was used according to the manufacturer's instructions. After the pull-down reaction was performed using GST–PAK–PBD beads, the precipitated proteins bound to the beads were analyzed by immunoblotting with anti-RAC1 or anti-CDC42 monoclonal antibody. Total RAC1 and CDC42 protein levels were assessed by Western blot analysis of total cell lysates.

The RHO Activation Assay Kit (Beyotime Institute of Biotechnology, Haimen, China) was used according to the manufacturer's instructions. After the pull-down reaction was performed using Rhotekin-RBD Agarose, the precipitated proteins bound to the beads were analyzed by immunoblotting with anti-RHOA monoclonal antibody. Total RHOA protein level was assessed by Western blot analysis of total cell lysates.
